# An expanded toolkit of drug resistance cassettes for *Candida glabrata*, *Candida auris*, and *Candida albicans* leads to new insights into the ergosterol pathway

**DOI:** 10.1128/msphere.00311-23

**Published:** 2023-11-06

**Authors:** Justin B. Gregor, Victor A. Gutierrez-Schultz, Smriti Hoda, Kortany M. Baker, Debasmita Saha, Madeline G. Burghaze, Cynthia Vazquez, Kendra E. Burgei, Scott D. Briggs

**Affiliations:** 1 Department of Biochemistry, Purdue University, West Lafayette, Indiana, USA; 2 Purdue University Institute for Cancer Research, West Lafayette, Indiana, USA; University of Georgia, Athens, Georgia, USA

**Keywords:** CRISPR-RNP, *Candida auris*, *Candida glabrata*, *Candida albicans*, *ERG5 *and* ERG3*, antifungal drug resistance, *KanMX *and* BleMX*, ergosterol pathway, toxic sterols, azole antifungal drugs

## Abstract

The World Health Organization recently published the first list of priority fungal pathogens highlighting multiple *Candida* species, including *Candida glabrata*, *Candida albicans*, and *Candida auris*. However, prior studies in these pathogens have been mainly limited to the use of two drug resistance cassettes, *NatMX* and *HphMX*, limiting genetic manipulation capabilities in prototrophic laboratory strains and clinical isolates. In this study, we expanded the toolkit for *C. glabrata*, *C. auris*, and *C. albicans* to include *KanMX* and *BleMX* when coupled with an *in vitro* assembled CRISPR-Cas9 ribonucleoprotein (RNP)-based system. Repurposing these drug resistance cassettes for *Candida*, we were able to make single gene deletions, sequential and simultaneous double gene deletions, epitope tags, and rescue constructs. We applied these drug resistance cassettes to interrogate the ergosterol pathway, a critical pathway for both the azole and polyene antifungal drug classes. Using our approach, we determined for the first time that the deletion of *ERG3* in *C. glabrata*, *C. auris,* and *C. albicans* prototrophic strains results in azole drug resistance, which further supports the conservation of the Erg3-dependent toxic sterol model. Furthermore, we show that an *ERG5* deletion in *C. glabrata* is azole susceptible at subinhibitory concentrations, suggesting that Erg5 could act as an azole buffer for Erg11. Finally, we identified a synthetic growth defect when both *ERG3* and *ERG5* are deleted in *C. glabrata,* which suggests the possibility of another toxic sterol impacting growth. Overall, we have expanded the genetic tools available to interrogate complex pathways in prototrophic strains and clinical isolates.

**IMPORTANCE:**

The increasing problem of drug resistance and emerging pathogens is an urgent global health problem that necessitates the development and expansion of tools for studying fungal drug resistance and pathogenesis. Prior studies in *Candida glabrata*, *Candida auris*, and *Candida albicans* have been mainly limited to the use of *NatMX/SAT1* and *HphMX/CaHyg* for genetic manipulation in prototrophic strains and clinical isolates. In this study, we demonstrated that *NatMX/SAT1, HphMX, KanMX,* and/or *BleMX* drug resistance cassettes when coupled with a CRISPR-ribonucleoprotein (RNP)-based system can be efficiently utilized for deleting or modifying genes in the ergosterol pathway of *C. glabrata*, *C. auris*, and *C. albicans*. Moreover, the utility of these tools has provided new insights into *ERG* genes and their relationship to azole resistance in *Candida*. Overall, we have expanded the toolkit for *Candida* pathogens to increase the versatility of genetically modifying complex pathways involved in drug resistance and pathogenesis.

## INTRODUCTION

Fungal infections pose a significant public health concern, with over a billion superficial infections and 1.5 million deaths, mostly from invasive infections, occurring annually worldwide (1, 2). *Candida* species are responsible for roughly 40%–70% of invasive fungal infections (1–3), and several species are classified as “high priority fungal pathogens” by the World Health Organization (WHO) for study, including *Candida glabrata*, *Candida albicans*, and *Candida auris*. Infections can range from superficial to life-threatening, with invasive candidiasis leading to a mortality rate of 20%–60% (4, 5). Currently, there are three FDA-approved major antifungals clinically used for the treatment of systemic fungal infections: azoles, echinocandins, and polyenes (6–8). However, antifungal drug resistance has become a significant concern, highlighted by the increase in clinically acquired drug resistance in *C. albicans* and *C. glabrata* and the recent emergence of a multi-drug-resistant pathogen, *C. auris* (8, 9).

Two of these drug classes, azole and polyenes, target the ergosterol pathway, an essential but complex biological pathway that is largely conserved across yeast and fungi (10–13). Most of our understanding of the ergosterol pathway has been derived from studies in auxotrophic *Saccharomyces cerevisiae* and *C. albicans* strains (13–15), whereas in comparison, our understanding of this pathway in *C. glabrata* and *C. auris* has been limited. These genetic and biochemical studies have proposed a model where azoles prevent ergosterol biosynthesis by directly inhibiting Erg11, the lanosterol 14-α-demethylase. Consequently, azole inhibition results in the production of a toxic sterol intermediate, 14α-methyl-3,6-diol, generated by the sterol Δ^5,6^-desaturase, Erg3 (14). Concurring with this model is the observation that deletion of *ERG3* in *S. cerevisiae* and *C. albicans* prevents the formation of the 14α-methyl-3,6-diol toxic sterol, resulting in azole resistance (14, 15). However, it has been reported and widely accepted in the field that deletion of *ERG3* in *C. glabrata* is not azole resistant but in contrast azole susceptible (16–18). Contrary to this observation, clinical isolates of *C. glabrata* and micro-evolved *ERG3* mutations can result in azole resistance, thereby providing an unexplained role for *ERG3* in *C. glabrata* (19, 20). Currently, it is unknown if an *ERG3* deletion results in azole resistance in *C. auris*. However, to further characterize the ergosterol pathway in prototrophic strains of *C. glabrata*, *C. auris*, and *C. albicans,* additional genetic tools are needed.

Currently, the *C. glabrata*, *C. auris*, and *C. albicans* toolkit has been mainly limited to the use of nourseothricin (*NatMX*/*SAT1*) or hygromycin B (*HphMX*/*CaHyg*). In contrast, drug resistance cassettes such as *KanMX* and *BleMX* are either rarely or not used in *Candida* but are commonly used in *Saccharomyces cerevisiae*. One major reason for this limitation is that *Candida* species have developed resistance to antibiotics, including kanamycin/geneticin (G418) and phleomycin/zeocin, which results in high background growth and prevents the ability to identify colonies containing *KanMX* and *BleMX* cassettes. The ability to repurpose and efficiently use these dominant selection markers in *Candida* would significantly enhance the flexibility for genetic manipulation of the ergosterol and/or other complex pathways when using prototrophic strains and clinical isolates.

In this study, we demonstrate that multiple drug resistance markers can be effectively used in *Candida* species when coupled with an *in vitro* assembled CRISPR-Cas9 ribonucleoprotein (RNP)-based system. Using this approach, we were able to efficiently make *erg3* deletions when using *NatMX*, *HphM*X, *KanMX*, or *BleMX* cassettes for *C. glabrata,* while *KanMX* and *BleMX* were used to make *erg3* deletions in *C. auris*. In addition, endogenous epitope tagging of *ERG3* and *ERG11* in *C. glabrata* was done using a 3×HA-*KanMX* cassette. Subsequently, the *ERG3* epitope-tagged gene was used to generate an *ERG3*-3×HA-*KanMX* cassette to functionally complement *erg3Δ* phenotypes. In addition, double gene deletions (*erg3Δerg5Δ*) were sequentially generated using *NatMX* and *HphMX* cassettes in *C. glabrata*. Finally, the ability to make simultaneous deletions of *C. albicans* alleles with a combinatorial drug selection approach using *CaKan*MX and *SAT1* or *BleMX* and *SAT1* cassettes was also demonstrated.

Our approach allowed us to not only enhance the *Candida* toolbox but also provide the genetic capability to efficiently manipulate the ergosterol pathway using prototrophic strains. Using these tools, we showed for the first time that *erg3Δ* strains are azole resistant in prototrophic *C. glabrata* strains, which provides support that the toxic sterol model is conserved in *C. glabrata*. In addition, prototrophic *C. auris* and *C. albicans erg3Δ* strains also showed an azole-resistant phenotype, demonstrating that the function of Erg3 is broadly conserved across all three pathogens. We also determined that an *erg5Δ* strain was susceptible to subinhibitory concentrations of azoles and suggested that Erg5 plays a role in azole buffering in *C. glabrata*. Finally, a synthetic growth defect was observed for a *C. glabrata erg3Δerg5Δ* double deletion strain, and we propose that this growth inhibition is caused by another toxic sterol. Overall, we have identified new insights involving the ergosterol pathway, and we anticipate that this expanded toolkit will provide the field the capacity to interrogate other complex pathways.

## RESULTS

### Short homology regions can be used for gene replacement in *C. glabrata* when coupled with CRISPR-RNP

**Fig 1:**
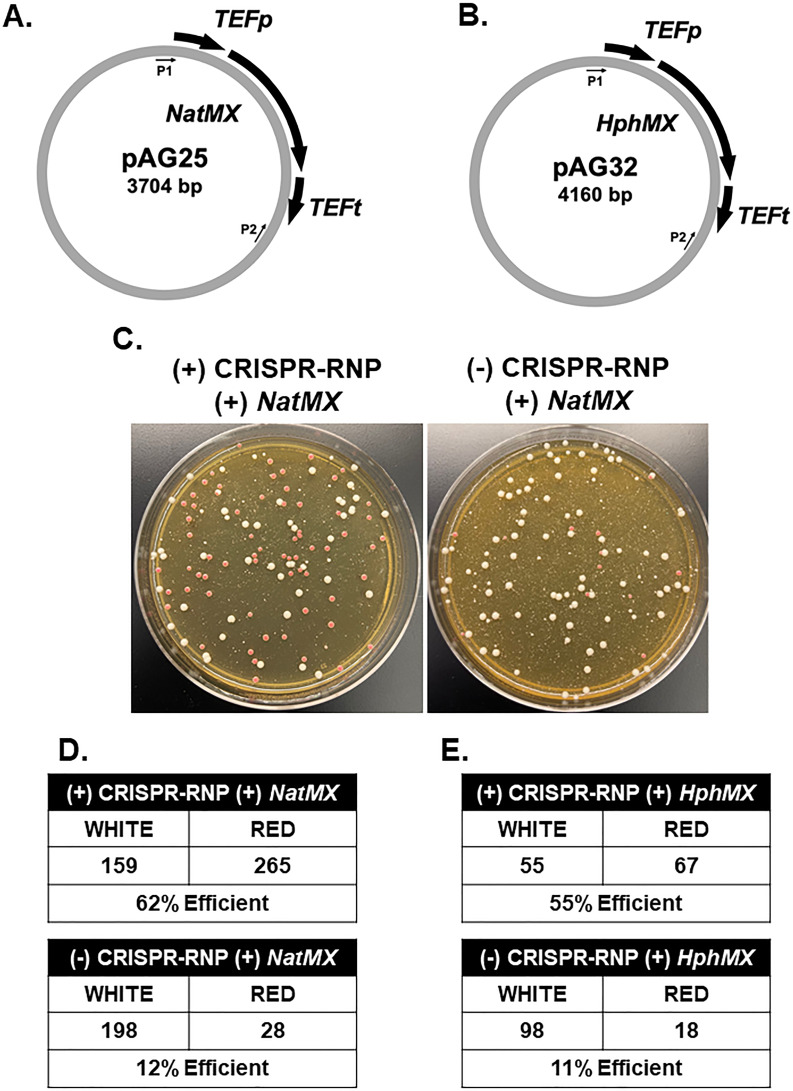
Homology regions of 130–150-bp efficiently generate *ADE2* deletions in *C. glabrata* using *NatMX* and *HphMX* when coupled with CRISPR-Cas9 RNP. (**A**) Schematic of the pAG25 *NatMX* plasmid. P1 and P2 indicate the locations of amplification sequences. (**B**) Schematic of the pAG32 *HphMX* plasmid. P1 and P2 indicate the locations of amplification sequences. (**C**) Representative transformation plate for *ADE2* deletion using *NatMX* with and without the addition of CRISPR-RNP. (**D**) Total number of positive transformants using *NatMX* with and without the addition of CRISPR-RNP. Numbers represent the summation across three separate transformations. (**E**) Total number of positive transformants using *HphMX* with and without the addition of CRISPR-RNP. Numbers represent the summation across three separate transformations.

CRISPR-mediated or non-CRISPR-based methods generally rely on large flanking homology regions ranging from 500 to 1,000 base pairs (bp) for efficient gene replacement in *Candida glabrata* (21, 22). Often, steps to generate long flanking regions are time-consuming and tedious using either cloning or multi-step fusion PCR approaches. The initial CRISPR-Cas9 RNP system developed for *Candida* species, including *C. glabrata,* utilized long homology regions ranging from 500 to 1,000 bp (21). However, it has been reported for *C. glabrata* that flanking homology regions ranging from 20 to 200 bp can be used for gene insertions resulting in gene disruption, albeit with the aid of a CRISPR-Cas9 plasmid-based system in an auxotrophic strain (23). To determine if short homology regions (HRs) flanking drug resistance cassettes were efficient in making gene deletions in *C. glabrata* using a CRISPR-Cas9 RNP method, we PCR amplified drug resistance cassettes using oligonucleotides (IDT Ultramers) of ~130–150 bp of homology to the *ADE2* gene. *ADE2* was selected due to its red pigment phenotype when the *ADE2* gene is disrupted, which allows for an unbiased determination of gene replacement efficiency (24–26). Using the pAG25 *NatMX* and pAG32 *HphMX* plasmids (Fig. 1A and B) (27), we deleted the *ADE2* open reading frame and counted the proportion of white and red colonies (Fig. 1C). With the addition of a CRISPR-Cas9 RNP containing two gRNAs and 130–150 bp of flanking homology, we observed a fivefold increase in the proportion of red colonies compared to the cassette alone (Fig. 1D). Similarly, we observed a fivefold increase in the proportion of red colonies using hygromycin B (*HphMX*) when using CRISPR-Cas9 RNP (Fig. 1E). With this efficiency, we determined that 500–1,000-bp homology regions are not required for efficient gene replacement in *C. glabrata*. To determine the lower limit of homology, we also tested 60-bp HRs and observed a 35% decrease in efficiency when compared to 130-bp HRs (Fig. S1A; Fig. 1D). Based on this comparison, we selected 130–150-bp HRs as the standard length for our CRISPR-RNP-mediated genetic manipulation. Altogether, these data suggest that short HRs are efficient in generating gene deletions in *C. glabrata* using *NatMX* and *HphMX* when coupled to CRISPR-RNP.

### Deletion of *ERG3* results in an azole drug-resistant phenotype in *C. glabrata*

We next applied our optimized approach to investigate the ergosterol pathway, a critical biosynthesis pathway targeted by both the azole and polyene antifungal drug classes. Azole drugs inhibit Erg11, lanosterol 14-α-demethylase, to block ergosterol biosynthesis, which leads to accumulation of an Erg3-dependent toxic sterol 14α-methyl-3,6-diol and growth inhibition (14, 28, 29) (see Fig. S2). While *ERG3* is known to have an azole-resistant phenotype when deleted or mutated in *S. cerevisiae* or *C. albicans* (14, 15, 30), *C. glabrata* auxotrophic strains deleted for *ERG3* show fluconazole susceptibility (16, 17). In contrast, micro-evolved *ERG3* mutations and clinical isolates show resistance to fluconazole (19, 20, 29). To address this unexplained contradiction, we used our optimized CRISPR-RNP method to make *erg3Δ* strains in the *Cg*2001 background strain using pAG25-*NatMX* and pAG32-*HphMX* as templates (Fig. 1A and B) and reported replacement efficiencies similar to those observed when targeting *ADE2* (Fig. S1B). In addition, the use of 60-bp HRs for generating *ERG3* deletions also showed 79% decreased efficiency when compared to 130-bp HRs (Fig. S1C).

**Fig 2:**
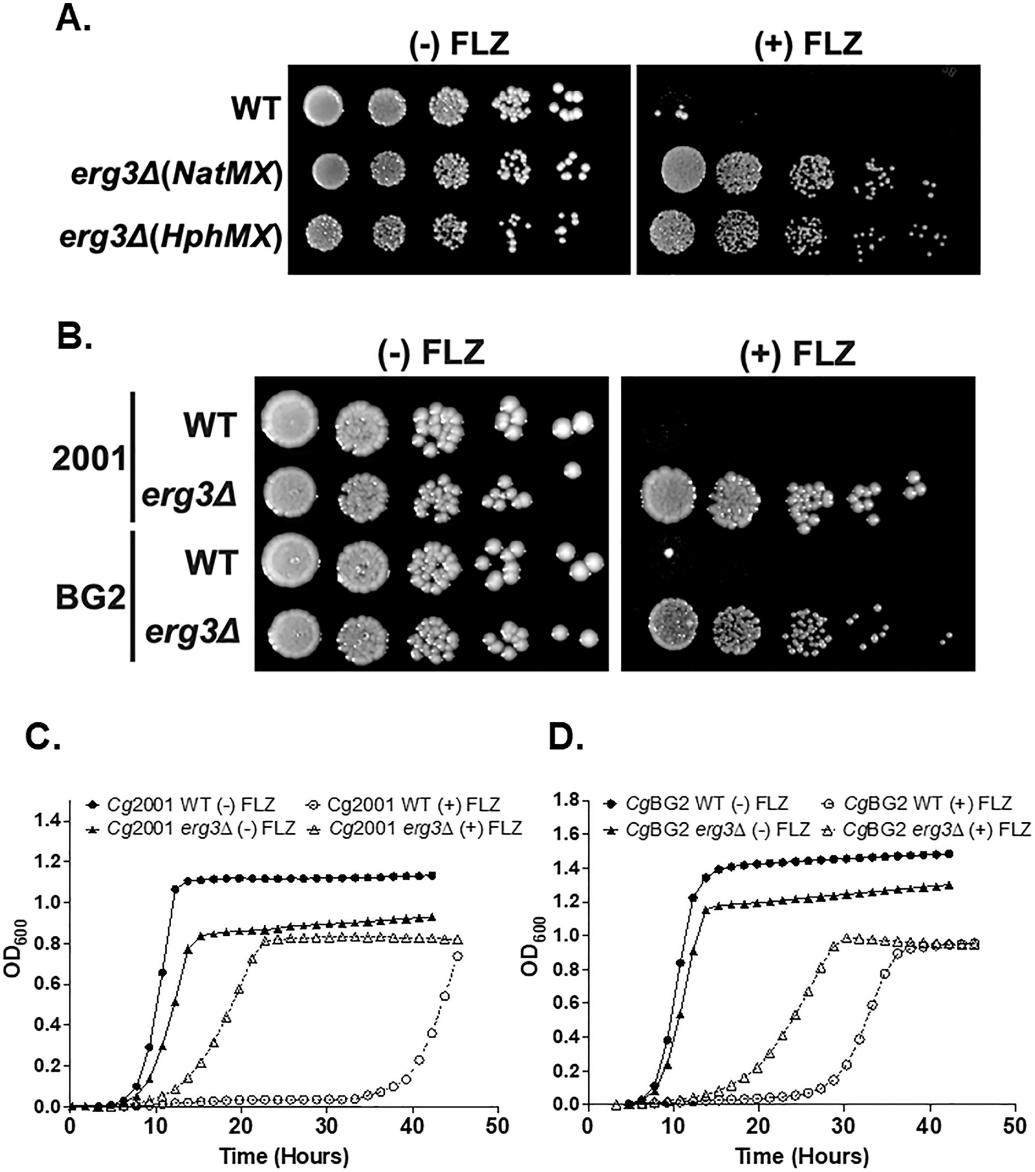
Deletion of *ERG3* results in an azole drug-resistant phenotype in *C. glabrata*. (**A and B**) Fivefold serial dilution spot assays with and without 64 µg/mL fluconazole (FLZ) in SC (A) and YPD (B) media. Indicated deletion strains were generated using CRISPR-Cas9 RNP. Images were captured at 48 hours. (**C and D**) Liquid growth assays of the indicated strains in YPD over 50 hours with and without 64 µg/mL FLZ, respectively. Growth curves represent the average of three biological replicates per strain.

After the *erg3Δ* strains were confirmed by PCR, we performed spot assays to confirm and compare their phenotypes with and without 64 µg/mL fluconazole. Both *erg3Δ* strains demonstrate a slow growth phenotype but also a clear increased resistance to fluconazole, in contrast to the previously published *erg3Δ* phenotypes in auxotrophic *C. glabrata* strains (Fig. 2A). To test whether this phenotype was strain specific, we deleted *ERG3* in the BG2 strain with CRISPR-RNP and performed spot assays with and without 64 µg/mL fluconazole and observed a similar azole-resistant phenotype to the *Cg*2001 *erg3Δ* strain (Fig. 2B). To quantify this difference, we performed liquid growth assays in both strains. We selected 64 µg/mL fluconazole as it led to a significant growth delay in both WT strains (Fig. S3A). Under untreated conditions, we observed minor differences in both doubling time and growth delays when comparing each *erg3Δ* strain with their parent WT strain (Fig. 2C and D; Fig. S3B). However, under fluconazole treatment, both *erg3Δ* strains had a significantly shorter growth lag compared to their respective WT, corroborating the plate-based assay results (Fig. 2C and D; Fig. S3C). Altogether, our data show that azole resistance does occur in prototrophic *Cg*2001 and BG2 strains when deleted for *ERG3*, suggesting that the function of Erg3 under azole treatment is conserved in *C. glabrata*.

### *ERG3* and *ERG5* deletions alter growth and azole drug susceptibility in *C. glabrata*

**Fig 3:**
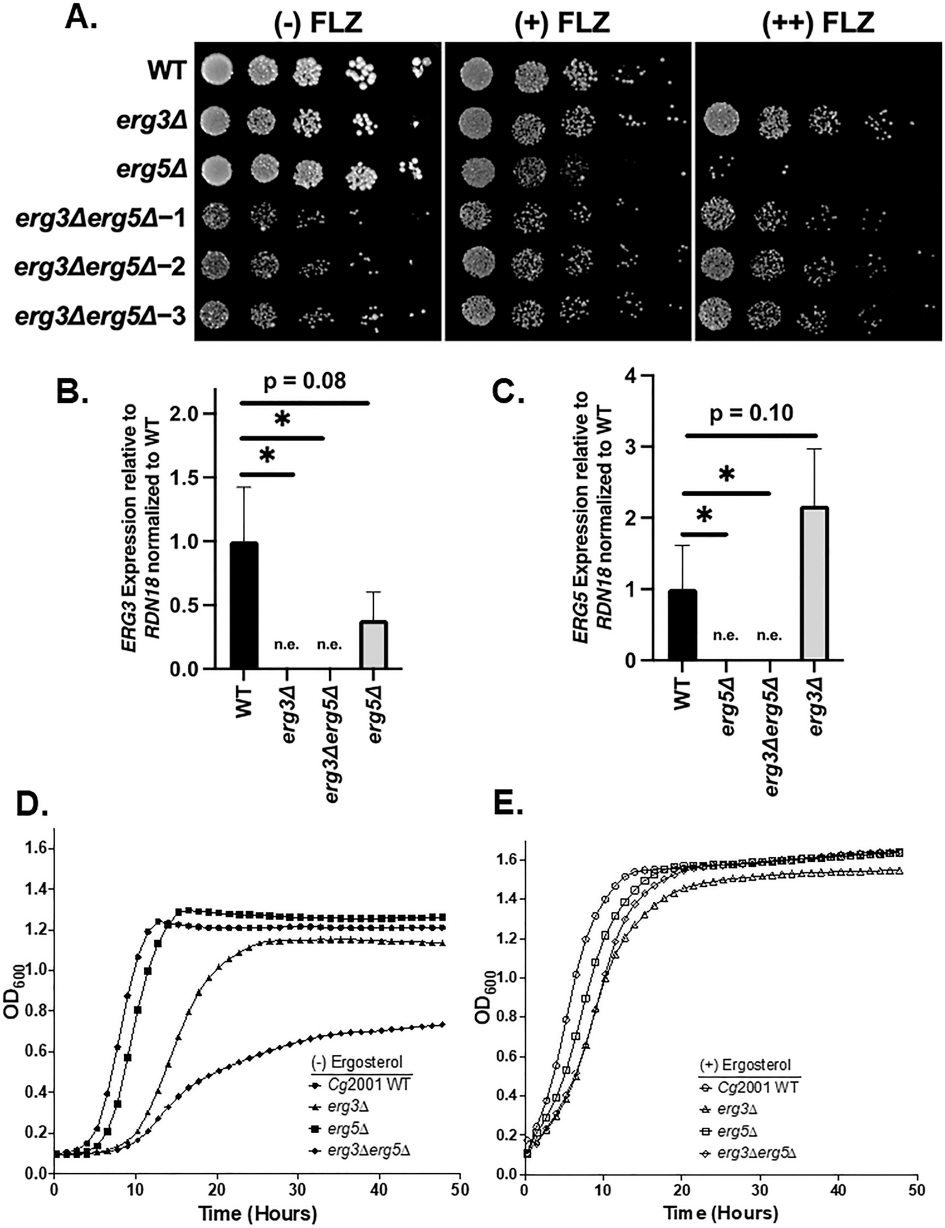
Double deletion of *ERG3* and *ERG5* results in a synthetic growth defect in *C. glabrata*. (**A**) Fivefold serial dilution spot assays with 0, 32, and 64 µg/mL fluconazole (FLZ) in SC media. Indicated deletion strains were generated using CRISPR-Cas9 RNP. (**B and C**) Expression of the indicated genes was determined by qRT-PCR analysis of mid-log phase cells in SC media. Data were normalized to *RDN18* mRNA levels and are the average of three biological replicates with three technical replicates each. Error bars represent the standard deviation. *P-value < 0.05. n.e., not expressed. Statistical analysis for qRT-PCR analysis was done using GraphPad PRISM using an unpaired two-tailed Student’s *t*-test. (**D and E**) Liquid growth assays of the indicated strains over 50 hours with and without 20 µg/mL ergosterol in SC media. Growth curves represent the average of three biological replicates per strain.

Similar to Erg11 (CYP51), Erg5 is also a known cytochrome P-450 (CYP61), which can be inhibited by azole drugs *in vitro* when using purified Erg5 protein from *S. cerevisiae* and *C. glabrata* at similar affinities to Erg11 (31–33). In addition, *Candida albicans*, *Neurospora crassa,* and *Fusarium verticillioides* fungal species deleted for *ERG5* show susceptibility to azoles, which has led to a hypothesis that Erg5 may serve as an azole buffer for Erg11 in these fungal species (34, 35). To determine the role of Erg5 in growth and azole susceptibility in *C. glabrata*, we generated an *erg5Δ* strain using our CRISPR-RNP approach. The *erg5Δ* strain grew similar to WT but showed susceptibility to 32 µg/mL fluconazole (Fig. 3A), which may be a consequence of more fluconazole inhibiting Erg11, indicating a protective buffering role for Erg5. Alternatively, under subinhibitory concentrations of azoles, an *erg5Δ* strain would produce Ergosta 5,7 dienol and/or 14α-methyl-3,6-diol, and both could be acting as growth inhibitory sterols (Fig. S2).

To determine if Ergosta 5,7 dienol is contributing to azole susceptibility, we generated an *erg3Δerg5Δ* double deletion strain in a previously constructed *erg3Δ* background. Based on known genetic and biochemical data (16, 36), the *erg3Δerg5Δ* double deletion would prevent the production of Ergosta 5,7 dienol (Fig. S2). While the pAG25 *NatMX* and pAG32 *HphMX* drug cassettes are effective for use in single deletions, it can be difficult to generate double gene deletions with drug cassettes when using non-CRISPR methods and short homology regions. This is particularly an issue when drug cassettes share similar flanking sequences, such as the *AgTEF1* promoter and the *AgTEF1* terminator (Fig. 1A and B). In this case, any subsequent gene deletion attempts could replace the drug cassette of the initially deleted gene, leading to drug cassette swapping. To circumvent this issue, we used our CRISPR-RNP approach and generated *erg3Δerg5Δ* strains using *HphMX* and *NatMX* resistance cassettes, which share similar promoter and terminator sequences. Despite the large *AgTEF1* homology regions present, we were able to efficiently generate *erg3Δerg5Δ* strains with the aid of CRISPR-Cas9-RNP (Fig. S1B). For strain confirmation, we performed qRT-PCR and detected no *ERG3* transcript in each strain lacking *ERG3* and no *ERG5* transcript in each strain lacking *ERG5* (Fig. 3B and C). Interestingly, we see a trend of decreased *ERG3* expression in the *erg5Δ* strain and increased expression of *ERG5* in the *erg3Δ* strain (Fig. 3B and C; Table S5). Although not statistically significant, this observation is consistent with what is observed in *S. cerevisiae* (37, 38). After confirming gene deletions by PCR and qRT-PCR analysis, spot assays were performed with and without fluconazole. Interestingly, all *erg3Δerg5Δ* strains suppressed azole susceptibility of *erg5Δ,* suggesting that 14α-methyl-3,6-diol and not Ergosta 5,7 dienol is contributing to *erg5Δ*’s susceptibility to azoles. Overall, our data support the hypothesis that azole susceptibility of *erg5Δ* with subinhibitory concentrations of azoles is a consequence of losing Erg5’s azole buffering effect, thereby allowing more azoles to inhibit Erg11.

To our surprise, all *erg3Δerg5Δ* strains display a synthetic growth defect, more than what was observed in the single *erg3Δ* and *erg5Δ* strains (Fig. 3A). We suspect that growth inhibition is due to the buildup of Ergosta 7-enol (Fig. S2). Despite this significant growth defect under untreated conditions, *erg3Δerg5Δ* strains grew on fluconazole-containing plates similar to an *erg3Δ* strain, which further supports the hypothesis that Ergosta 7-enol and 14α-methyl-3,6-diol are acting as growth inhibitory toxic sterols in *C. glabrata*.

In addition to plate-based growth assays, we performed liquid growth assays to quantify changes in growth. The *erg5Δ* strain had a doubling time and lag phase similar to WT. The *erg3Δ* strain had a growth defect with a twofold longer doubling time and lag phase compared to WT (Fig. 3D; Fig. S4). The *erg3Δerg5Δ* strain had a synthetic growth defect with a 3.5-fold increase in both doubling time and lag phase compared to WT, as well as a decrease in OD_600_ saturation (Fig. 3D; Fig. S4). We hypothesized that these defects were due to altered sterol content in the *erg3Δ* and *erg3Δerg5Δ* strains, so we tested if supplementation with 20 µg/mL ergosterol could rescue these phenotypes. Strikingly, ergosterol supplementation led to a near complete rescue of the growth delays in each *ERG* deletion strain as well as a complete rescue of the saturation defect in *erg3Δerg5Δ* strains (Fig. 3E). Additionally, *erg3Δ* strains grew better in YPD vs SC media, which we suspect is due to the presence of ergosterol in YPD but not in SC media (Fig. 2C vs 3D), further supporting that the growth defects of *ERG* deletion strains are likely due to altered sterol content.

Overall, our genetic studies have identified a potential azole buffering effect for Erg11 contributed by Erg5. In addition, when both *ERG3 and ERG5* are deleted, we observe a synthetic growth defect that is likely caused by the buildup of Ergosta 7-enol, suggesting that this sterol is acting as a toxic sterol that inhibits growth. Additional biochemical and genetic studies will be needed to fully understand the observed *ERG* gene deletion phenotypes.

### *BleMX* and *KanMX* can be used efficiently to make *ADE2* and *ERG3* deletions in *C. glabrata* when using CRISPR-RNP, where *ERG3* deletion results in azole drug resistance

**Fig 4:**
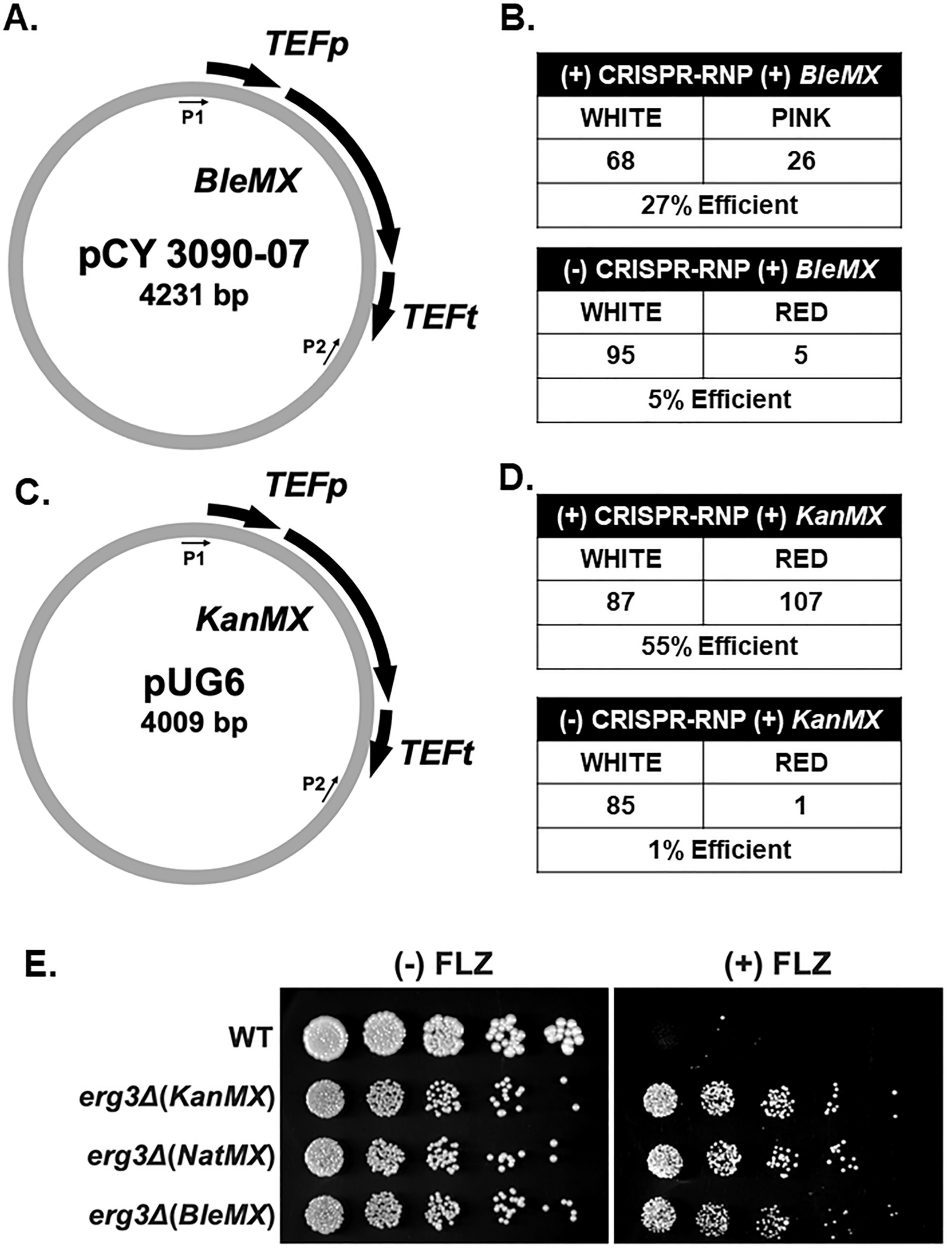
*BleMX* and *KanMX* can be used as efficient drug resistance cassettes in *C. glabrata* when coupled with CRISPR-Cas9 RNP. (**A**) Schematic of the pCY3090-07 plasmid. P1 and P2 indicate the locations of amplification primer sequences. (**B**) Total number of positive transformants using *BleMX* with and without the addition of CRISPR-Cas9 RNP. Numbers are the summation across three separate transformations. (**C**) Schematic of the pUG6 plasmid. P1 and P2 indicate the locations of amplification primer sequences. (**D**) Total number of positive transformants using *KanMX* with and without the addition of CRISPR-RNP. Numbers are the summation across three separate transformations. (**E**) Fivefold serial dilution spot assays of indicated strains with and without 64 µg/mL fluconazole (FLZ) in SC media. Images were captured at 48 hours.

To further investigate the ergosterol pathway in prototrophic strains, additional drug resistance markers are needed. Since our CRISPR-RNP system is efficient at generating single and double deletions in *C. glabrata* using *NatMX* and *HphMX*, we then tested whether this system was effective for using other drug resistance cassettes typically not used in *C. glabrata*. We first tested *BleMX*, which confers resistance to zeocin, as the use of *BleMX* has been reported once in *C. glabrata* using a non-CRISPR transformation method, albeit at extremely low efficiency (<1%) (39). To first determine whether the CRISPR-Cas9 RNP system effectively generates gene deletions using *BleMX*, we deleted the entire open reading frame of *ADE2* using pCY3090-07 as a template (Fig. 4A) (40). When comparing the proportion of red colonies with and without the addition of CRISPR-Cas9, a five- to sixfold increase in efficiency was observed when using CRISPR (Fig. 4B).

Next, we tested whether this system permitted efficient use of *KanMX* as a drug resistance cassette in *C. glabrata*. Although *KanMX* is routinely used in *S. cerevisiae*, *KanMX* has not been successfully utilized for genetic manipulations in *C. glabrata*, with the exception of one study (41). Effective use of *KanMX* would allow for direct repurposing of many *S. cerevisiae* tagging and deletion *KanMX* cassettes for *C. glabrata*. To test this, we deleted *ADE2* using a *KanMX* drug resistance cassette amplified from pUG6 (Fig. 4C) (42). With the addition of CRISPR-RNP, we observed a 55-fold increase in efficiency, suggesting that CRISPR-RNP is required for the use of *KanMX* in *C. glabrata* (Fig. 4D).

We also deleted *ERG3* with *KanMX* or *BleMX* using CRISPR-RNP with similar efficiencies to *ADE2* (Fig. S1B). By spot assays, we observed an azole-resistant phenotype similar to the other constructed *erg3Δ* strains (Fig. 4E). These data demonstrate that *KanMX* and *BleMX* can be used as effective drug resistance cassettes in *C. glabrata* when coupled with CRISPR.

### The *KanMX* drug resistance cassette can be repurposed for generating endogenous epitope-tagged proteins in *C. glabrata*

**Fig 5:**
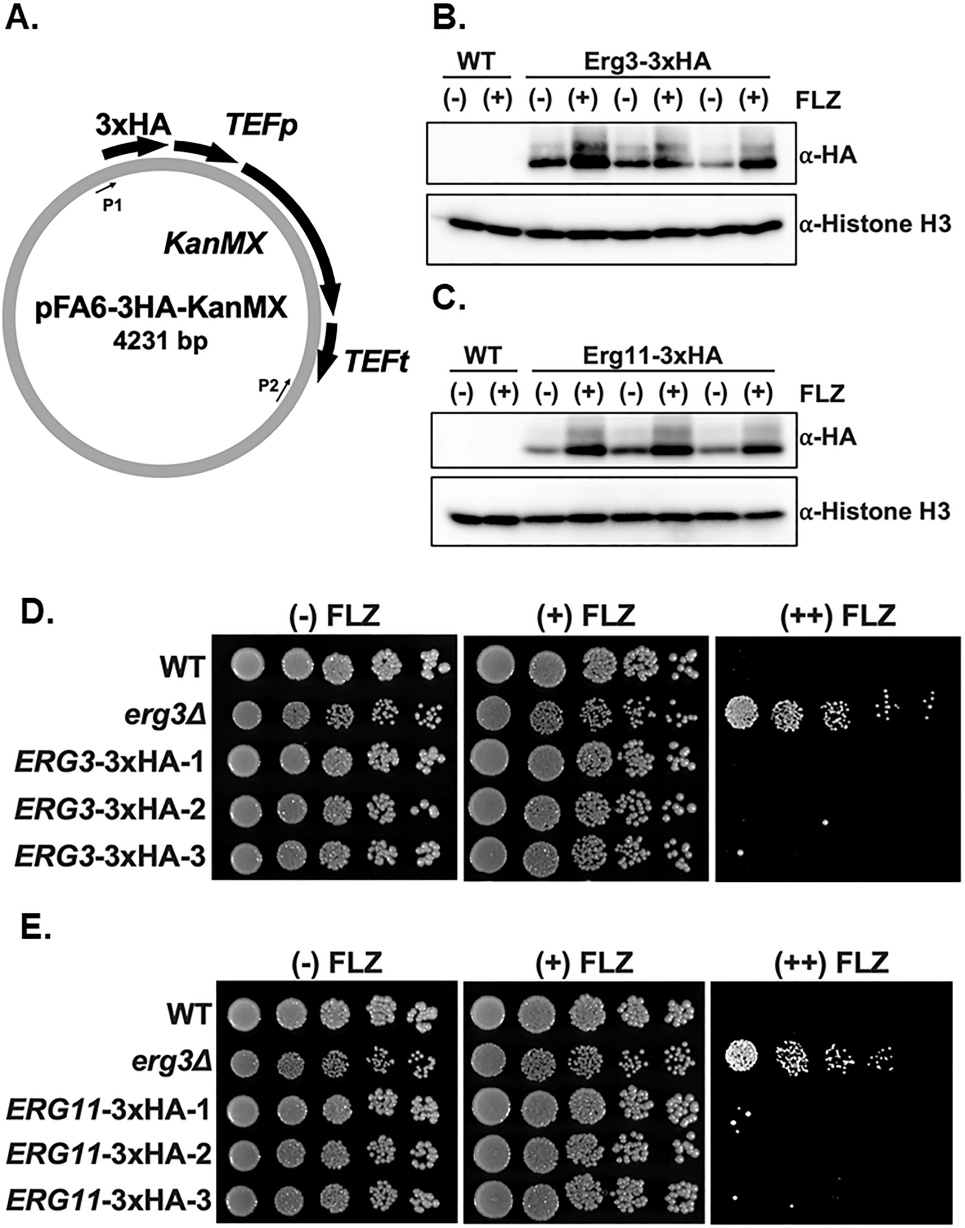
C-terminal 3×HA tagging of *ERG3* and *ERG11* does not alter azole susceptibility in *C. glabrata*. (**A**) Schematic of the pFA6-3HA-KanMX plasmid. P1 and P2 indicate the locations of amplification primer sequences. (**B and C**) Indicated strains were either untreated (−) or treated (+) with 64 µg/mL fluconazole (FLZ) for 3 hours. Whole cell extracts were isolated and immunoblotted against an anti-HA antibody for the detection of Erg3 or Erg11. Histone H3 was used as a loading control. Three independent clones were represented for Erg3-3×HA and Erg11-3×HA. (**D and E**) Fivefold serial dilution spot assays in SC media of indicated strains with 0, 16, and 64 µg/mL FLZ, respectively. Three independent clones were represented for Erg3-3×HA and Erg11-3×HA. Images were captured at 48 hours.

Because our data indicate that *KanMX* is a suitable drug resistance cassette for gene deletions in *C. glabrata*, we wanted to determine if *KanMX* could be used for endogenous epitope tagging of *ERG3* and *ERG11* using the C-terminal 3×HA-*KanMX* plasmid (pFA6a/PYM1) designed for *Schizosaccharomyces pombe* but also used in *S. cerevisiae* (Fig. 5A) (43, 44). *ERG3* and *ERG11* tags were generated using CRISPR-RNP and G418 selection. After PCR confirmation, tagged strains were grown with and without 64 µg/mL fluconazole in SC media and collected at mid-log phase for immunoblotting using anti-HA (12CA5). Histone H3 was used as a loading control. Our data indicate that Erg3 and Erg11 proteins are expressed under untreated conditions and induced under fluconazole treatment (Fig. 5B and C), which is consistent with transcript analysis from previous studies (45, 46). To confirm that the epitope tag does not alter function, we performed spot assays with and without 64 µg/mL fluconazole, using an *erg3Δ* strain as a control. All epitope-tagged Erg3-3×HA and Erg11-3×HA strains grow similar to WT under both untreated and fluconazole treatment (Fig. 5D and E). Altogether, these data suggest that *KanMX* epitope tagging constructs used in *S. pombe* and *S. cerevisiae* can be repurposed in *C. glabrata* and that C-terminal tagging of Erg11 and Erg3 does not alter their function.

### *ERG3-3×HA-KanMX* complements *erg3Δ* and *erg3Δerg5Δ* phenotypes

**Fig 6:**
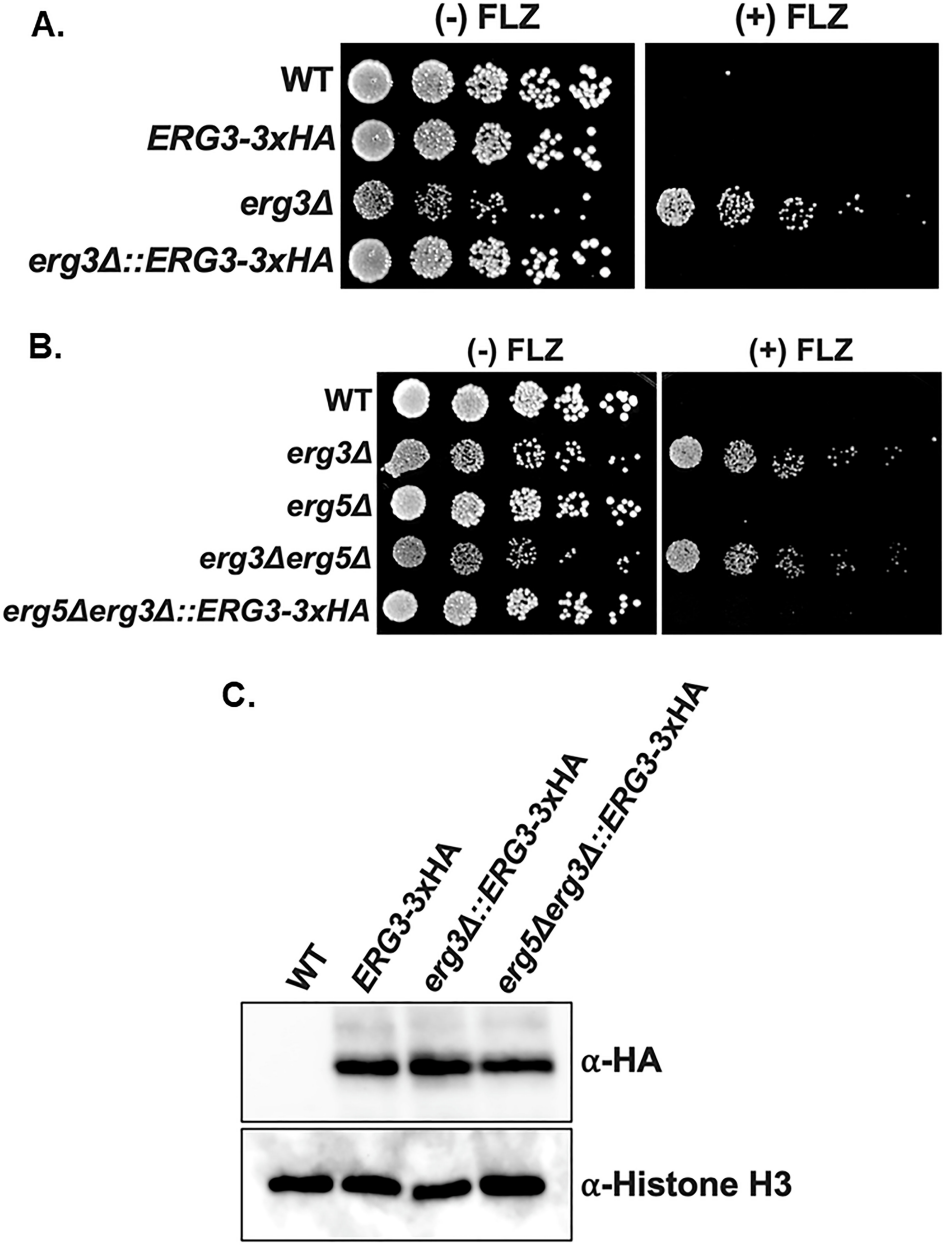
Complementation at *ERG3*’s endogenous locus with *ERG3*-3×HA-*KanMX* rescues *erg3Δ* and *erg3Δerg5Δ* phenotypes. (**A and B**) Fivefold serial dilution spot assays of indicated strains with 0 and 64 µg/mL fluconazole (FLZ) in SC media. Images were captured at 48 hours. (**C**) Whole cell extracts were isolated and immunoblotted against an anti-HA antibody for the detection of Erg3 in complemented strains. Histone H3 was used as a loading control, and *ERG3*-3×HA was used as a positive control.

Because C-terminal tagging of *ERG3* did not disrupt function (Fig. 5D), we used the *ERG3-3×HA-KanMX* strain as a genomic template to generate a replacement cassette to complement the *erg3Δ* and *erg3Δerg5Δ* strain phenotypes. The *ERG3-3×HA-KanMX* cassette was PCR amplified, and CRISPR-RNP was targeted to the *NatMX* cassette that was used to delete *ERG3*. Re-introducing *ERG3* at its endogenous locus in the *erg3Δ* strain restored WT growth and azole susceptibility (Fig. 6A). In addition, re-introduction of *ERG3* at its endogenous locus in the *erg3Δerg5Δ* strain reverts *erg3Δerg5Δ* growth back to *erg5Δ* and restores azole susceptibility (Fig. 6B). We were able to confirm that Erg3 is expressed by Western blotting (Fig. 6C). Altogether, these data support that the azole-resistant phenotype observed in our *erg3Δ* strains can be suppressed when complemented with Erg3. These complementation studies indicate that Erg3 was solely responsible for the *erg3Δ* azole-resistant phenotype. In addition, restoring Erg3 in *erg3Δ* and *erg3Δerg5Δ* strains rescues azole susceptibility, which is a consequence of Erg3’s ability to produce the growth inhibitory toxic sterol 14α-methyl-3,6-diol (Fig. S2). Furthermore, complementation of *erg3Δerg5Δ* also suppressed the growth defect, further supporting that Ergosta-7-enol is another potential growth inhibitory toxic sterol. Because of our demonstrated efficient use of *KanMX* and *BleMX*, additional genetic studies can be done to further address these discoveries in prototrophic strains.

### *BleMX* and *KanMX* can be used efficiently to make *ADE2* and *ERG3* deletions in *C. auris* when using CRISPR-RNP, where *ERG3* deletion results in azole drug resistance

**Fig 7:**
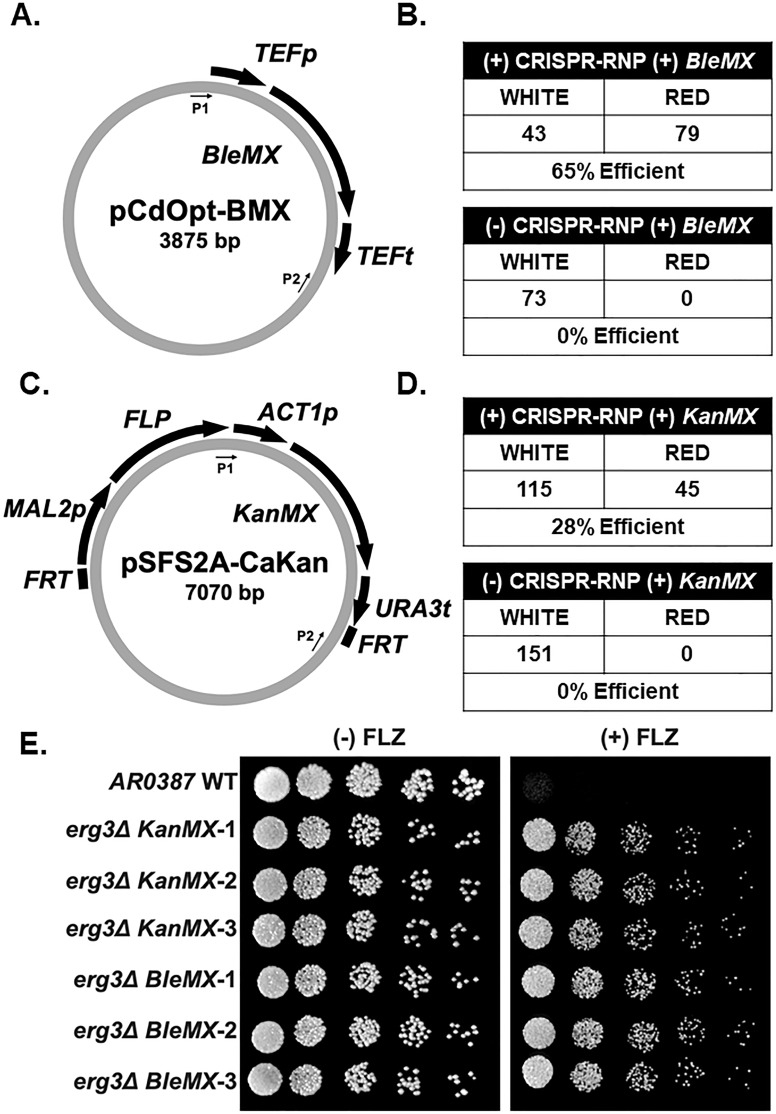
*BleMX* and *KanMX* can be used as drug resistance cassettes in *C. auris* when coupled with CRISPR-Cas9-RNP. (**A**) Schematic of the pCdOpt-BMX plasmid. P1 and P2 indicate the locations of amplification primer sequences. (**B**) Total number of positive transformants using *BleMX* with and without the addition of CRISPR-Cas9 RNP. (**C**) Schematic of the pSFS2A-CaKan plasmid. P1 and P2 indicate the locations of amplification primer sequences. (**D**) Total number of positive transformants using *KanMX* with and without the addition of CRISPR-Cas9 RNP. (**E**) Fivefold serial dilution spot assays of indicated *C. auris* strains with and without 64 µg/mL fluconazole (FLZ) in SC media. Three independent clones were represented for each *erg3Δ* strain deleted with *BleMX* or *KanMX*. Images were captured at 48 hours.

Next, we tested whether we could use *BleMX* and *KanMX* as drug resistance cassettes in the emerging pathogen, *C. auris*, since previous studies in *C. auris* have been limited to using *SAT1* and *CaHyg* as drug resistance cassettes (47, 48). We first generated a codon-optimized *BleMX* for use in CTG clade species and named the plasmid pCdOpt-BMX (Fig. 7A). Using this codon-optimized *BleMX* plasmid as a template, we deleted *ADE2* in *C. auris AR0387* using the CRISPR-RNP method and reported 65% efficiency compared to 0% replacement without CRISPR (Fig. 7B). Next, we used pSFS2A-CaKan codon-optimized *KanMX* as a template and again targeted *ADE2* (Fig. 7C). Using this codon-optimized *KanMX*, we report 29% efficiency with CRISPR compared to 0% without (Fig. 7D).

Because *erg3Δ* phenotypes have not been reported in *C. auris*, we deleted *ERG3* with both *BleMX* and *KanMX*. After PCR confirmation, we performed spot assays with and without 64 µg/mL fluconazole. Similar to the *C. glabrata erg3Δ* strains, we observed an azole-resistant phenotype across all clones tested (Fig. 7E). Altogether, these data demonstrate that *BleMX* and *KanMX* can be used for efficient gene replacement in *C. auris* when coupled with CRISPR. This is the first report that *erg3Δ* strains are azole resistant in *C. auris*, demonstrating the conservation of Erg3 function in *C. auris* and providing support for Erg3-dependent production of the toxic 14α-methyl-3,6-diol sterol.

### *BleMX* and *KanMX* can be used to make simultaneous *ERG3* allele deletions in *C. albicans* when coupled with *SAT1* and CRISPR-RNP

Because our data show that *KanMX* and *BleMX* are effective drug resistance cassettes for deleting *ERG3* in prototrophic *C. glabrata* and *C. auris* strains when coupled to CRISPR-RNP, we next tested if these drug resistance cassettes could be used in *C. albicans*. Although *erg3Δ* strains have been generated in auxotrophic *C. albicans* strains (15), homozygous *erg3Δ* strains have not been characterized in a prototrophic SC5314 strain or other prototrophic *C. albicans* isolates. In contrast to *C. glabrata* and *C. auris*, *C. albicans* possesses innate resistance toward G418, hygromycin B, and zeocin even when using high concentrations of these drug compounds, which prevents using them for genetic manipulation. Interestingly, a dual selection approach with both *SAT1* and *HphMX* replacement cassettes coupled with a CRISPR-RNP approach was shown to be effective at generating homozygous double deletions (49). Therefore, we hypothesized that an analogous approach using *SAT1* and *BleMX* or *KanMX* would work for deleting *ERG3*.

**Fig 8:**
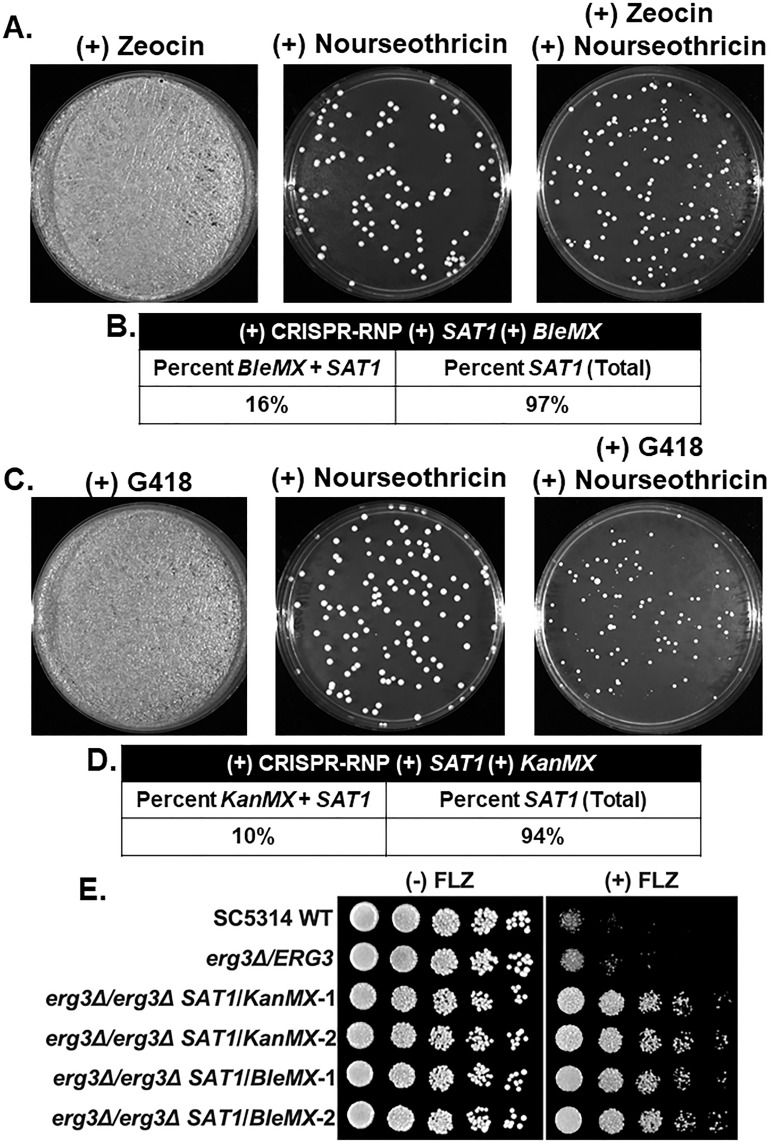
*BleMX* and *KanMX* can be used in conjunction with *SAT1* for simultaneous homozygous deletions in *C. albicans*. (**A**) Representative transformation plated on zeocin alone, nourseothricin alone, and both zeocin and nourseothricin. Images were captured at 48 hours. (**B**) Percentage of homozygous and heterozygous *ERG3* deletions screened by PCR from *BleMX+SAT1* double selection plates. (**C**) Representative transformation plated on G418 alone, nourseothricin alone, and both G418 and nourseothricin. Images were captured at 48 hours. (**D**) Percentage of homozygous and heterozygous *ERG3* deletions screened by PCR from *KanMX+SAT1* double selection plates. (**E**) Fivefold serial dilution spot assays of indicated *C. albicans* strains with and without 64 µg/mL fluconazole (FLZ) in SC media. Images were captured at 48 hours.

To test this approach, *ERG3* drug resistance cassettes were PCR amplified using both pCdOpt-BMX (*BleMX*) and pBBS2-*SAT1*-FLP as templates and simultaneously transformed into SC5314 using our CRISPR-RNP method. When transformants were plated on zeocin alone, we observed no drug selectivity; however, drug selectivity was observed on plates containing both zeocin and nourseothricin or nourseothricin alone (Fig. 8A). From the zeocin and nourseothricin plates, we obtained 16% positive *erg3Δ/erg3Δ* strains confirmed by PCR (Fig. 8B; Fig. S5A). We then used the same transformation approach but instead with *SAT1* and *KanMX* as replacement templates. Again, we observed no selectivity when plated on G418 plates alone, but drug selectivity was observed on plates containing both G418 and nourseothricin (Fig. 8C). However, we obtained 10% positive *erg3Δ/erg3Δ* strains, and PCR confirmed the integration of both cassettes (Fig. 8D; Fig. S5B). Interestingly, we were able to get >94% of the *SAT1* integration, which allowed us to identify heterozygous *ERG3/erg3Δ* strains, indicating that both homozygous and heterozygous deletions can be identified from colonies grown on the combined drug plate (Fig. 8B and D; Fig. S5A and B).

To assess the deletion strains, spot assays were performed with and without 64 µg/mL fluconazole (Fig. 8E). Azole resistance was observed for all *erg3Δ/erg3Δ* strains, corroborating previous deletions in auxotrophic *C. albicans* strains (15). Similar to *HphMX*, our data show that *BleMX* and *KanMX* can be used in conjunction with *SAT1* for homozygous double deletion engineering in *C. albicans*. In addition to *C. glabrata* and *C. auris*, we have also expanded the tools to genetically manipulate prototrophic *C. albicans* strains.

## DISCUSSION

In this study, we have expanded the *Candida* toolbox to include *KanMX* and *BleMX* when coupled to CRISPR-RNP, which has allowed us to efficiently manipulate the ergosterol pathway. Using these tools, we have established for the first time that deleting *ERG3* results in an azole drug-resistant phenotype in *C. glabrata*, *C. auris,* and *C. albicans* prototrophic strains. We also show that a *C. glabrata erg5Δ* strain is susceptible to azoles, while *erg3Δerg5Δ* strains show a synthetic growth defect. Overall, we provided new tools for genetic manipulation that allowed us to determine the impact of multiple *ERG* gene deletions in *Candida*.

In *S. cerevisiae* and *C. albicans*, *erg3Δ* strains have been well characterized to have an azole drug-resistant phenotype, which is attributed to the failure to make the growth inhibitory toxic sterol, 14α-methyl-3,6-diol (14, 15). In contrast, the prevailing thought in the field is that this does not occur when *ERG3* is deleted in *C. glabrata* because *erg3Δ* strains have been reported to be susceptible to azole drugs (16, 17). However, this observation has been based mainly on one report where an *ERG3* deletion in an auxotrophic *C. glabrata* strain was reported to be azole susceptible (16). Based on our findings, Erg3’s function is conserved, and deletion of *ERG3* leads to azole drug resistance in all prototrophic strains tested. Furthermore, our *ERG3* complemented strains generated by our expanded toolkit show that azole drug susceptibility can be restored, indicating that only *ERG3* was responsible for the observed phenotypes.

Currently, it is unclear why other groups have observed susceptibility in *C. glabrata* auxotrophic *erg3Δ* strains. However, an issue using auxotrophic strains for studying the ergosterol pathway could be an unintentional altered growth phenotype caused by mutations in the amino acid biosynthetic pathway. For example, it has been shown in *S. cerevisiae* that azole-resistant phenotypes in an *erg3Δ* strain were contingent on the auxotrophic status of the strain (50). Alternatively, because azoles have been shown to bind to Erg5 and Erg11 with equal affinity and inhibit both enzymes *in vitro* (31–33), the observed azole susceptibility difference in *erg3Δ* strains could be caused by differences in Erg5 expression or non-synonymous mutations across *C. glabrata* strains. These differences could alter the buffering capacity of Erg5 toward azoles and thus change the concentration of azoles that inhibit Erg11. Another possibility could be a paradoxical effect where subinhibitory concentrations of azoles in an *erg3Δ* strain fail to completely block Erg11, resulting in a potential buildup of another toxic sterol, leading to azole susceptibility. In this case, if Erg5 is effectively inhibited in *erg3Δ*, this would phenocopy an *erg3Δerg5Δ* strain, resulting in a buildup in Ergosta-7-enol and growth inhibition (Fig. 3D; Fig. S2). Further investigation will be needed to determine why the reported auxotrophic *erg3Δ* strains show azole susceptibility and whether this is due to amino acid uptake, Erg5 fluconazole buffering effect, and/or toxic sterol. Nonetheless, we observed broad conservation of *erg3Δ* phenotypes using prototrophic strains resulting in azole resistance across *C. glabrata*, *C. auris*, and *C. albicans* when treated with growth inhibitory azole concentrations, supporting the notion that Erg3 contributes to the production of the toxic sterol 14α-methyl-3,6-diol in all prototrophic strains tested.

In this study, we show that two additional drug resistance cassettes, *KanMX* and *BleMX*, commonly used for *S. pombe* or *S. cerevisiae* can be repurposed and used reliably in *C. glabrata*. Moreover, we show that the codon-optimized *KanMX* from the pSFS2A-CaKan (*KanMX*) plasmid (51) and our generated codon-optimized *BleMX* from the pCdOpt-BMX plasmid can be used for *C. auris*. In contrast to *C. glabrata* and *C. auris*, *KanMX* or *BleMX* in *C. albicans* cannot be used due to this organism’s high tolerance/resistance to the aminoglycoside antibiotic G418 and the glycopeptide-derived antibiotics bleomycin, phleomycin, and zeocin (see Fig. 6). Because the CRISPR-RNP-based system has been used successfully to simultaneously delete both alleles in *C. albicans* when using *SAT1* and *HygB* (49), we applied a similar approach to use *KanMX* and *BleM*X. While we observed lower percentages of homozygous double deletions using this approach, adjuvants such as quinine or molybdate have been shown to suppress background growth of *C. albicans* when grown on G418 or hygromycin, which allows successful integration of codon-optimized *CaKan* and *CaHygB* cassettes (51). The addition of adjuvants and/or the use of integrated/transient expression-based CRISPR systems (26, 52) could further improve the efficiency of simultaneous allele deletions.

Our study also successfully demonstrates the repurposing of *KanMX*-containing plasmids traditionally utilized for making gene deletions or C-terminal epitope tags in *S. pombe* or *S. cerevisiae* for use in *C. glabrata*. While we clearly demonstrate that the endogenous C-terminal 3×HA tagging constructs are suitable for *C. glabrata*, this approach may not work for all genes, as C-terminal tagging may disrupt the function of the protein. Thus, our approach would also allow for repurposing endogenous N-terminal *KanMX* tagging constructs designed for *S. cerevisiae* (53). Additionally, the efficiency of endogenous epitope-tagged proteins using CRISPR allows for more functional and mechanistic studies beyond transcript analysis, as endogenous epitope-tagged proteins have been used sparingly in prototrophic strains and clinical isolates of *C. glabrata*. This is particularly important since antibodies to endogenous proteins are scarce and costly to make. Finally, we show that endogenous epitope-tagged genes can be used as templates to complement the function of their respective gene deletion.

Overall, our study provides the field with additional ways to efficiently manipulate prototrophic *Candida* pathogens when using a CRISPR-based approach. Importantly, this approach provides us with further insight into the ergosterol pathway, although additional studies would be needed to address the mechanisms of our new observations. Applying this expanded toolkit in *Candida* should also enhance our understanding of other complex pathways impacting fungal drug resistance and pathogenesis.

## MATERIALS AND METHODS

### Yeast strains and plasmids

All strains used are described in Table S1. *C. glabrata* strains were derived from *Cg*2001 or BG2 (ATCC 2001). *C. albicans* strains were derived from SC5314 (54), a gift from William A. Fonzi, Georgetown University. The *C. auris* AR0387 strain was obtained from the CDC AR Isolate Bank. The pAG25, pAG32, and pUG6 plasmids were obtained from Euroscarf (27, 42). The pFA6a-3HA-*KanMX*, pCY3090-07, and pSFS2A-CaKan plasmids were obtained from Addgene (40, 43, 51). The pBSS2-*SAT1* flipper plasmid was provided to us by P. David Rogers, St. Jude Children’s Research Hospital, with permission from Joachim Morschauser (55). pCdOpt-BMX (*BleMX*) was synthesized by IDT, where the *TEF1p-BleMX-TEF1t* sequence was codon optimized for CTG clade *Candida* species, synthesized, and cloned into the pUCIDT plasmid. The pCdOpt-BMX plasmid can be obtained at Addgene (ID number 203929).

### PCR amplification for gene deletion and epitope tagging

All oligonucleotides used are denoted in Table S2. PCR amplification of drug resistance cassettes is as follows: 95°C for 5 minutes, 95°C for 30 seconds, 52°C for 30 seconds, and 72°C for 2–3 minutes for a total of 30 cycles, with a final elongation step at 72°C for 10 minutes. The PCR products were purified from agarose gels.

### CRISPR gRNA design and selection

Custom Alt-R CRISPR gRNAs were designed and ordered from Integrated DNA Technologies (Table S3). For each gene deletion, two CRISPR gRNAs were designed close to the 5′ and 3′ open reading frames (ORFs) of the gene of interest. For epitope tagging, one CRISPR gRNA was designed in the 3′ untranslated region (UTR) of the gene of interest. CRISPR gRNAs were selected based on their designated “on-target score” as determined by the CRISPR-Cas9 guide RNA design checker (IDT). Potential gRNAs were screened for off-target events using the CRISPR RGEN Tools Cas OFFinder (http://www.rgenome.net/cas-offinder/). Selected gRNAs required >75 on-target scores as well as 0 potential off-target events with three mismatches or less.

### CRISPR-Cas9 RNP system

The CRISPR-Cas9 RNP method was based on Grahl et al. with slight modifications (21). Briefly, Alt-R CRISPR crRNA and tracrRNA were used at a working concentration of 20 µM. The CRISPR-Cas9 crRNAs:tracrRNA hybrid was made by mixing together 1.6 µL of crRNA (8 µM final concentration), 1.6 µL of tracrRNA (8 µM final concentration), and 0.8 µL of RNAse-free water. For gene deletions, two crRNAs, 0.8 µL each, were added at a stoichiometric equivalent to tracrRNA, and for C-terminal tagging, one crRNA, 1.6 µL, was used. The CRISPR-RNP mix was incubated at 95°C for 5 minutes and allowed to cool to room temperature. Three microliters of 4 µM Cas9 (IDT) was added to the mix (final concentration of 1.7 µM) and incubated at room temperature for 5 minutes.

### Cell transformation

Twenty-five milliliters of the desired strain was grown to an OD_600_ of 1.6 to saturation prior to transformation. Cells were resuspended in 10 mL of 1× LiTE Buffer (100 mM LiAc, 10 mM Tris-HCl, and 1 mM EDTA) and shaken at 250 rpm at 30°C for an hour. DTT was added to a final concentration of 100 mM, and cells were incubated at 30°C for an additional 30 minutes. Cells were collected by centrifugation, washed twice with 1 mL of ice-cold water, and washed once with 1 mL of cold sorbitol. Cells were resuspended in 200 µL of cold sorbitol for electroporation.

### Electroporation and colony PCR

Twenty microliters of prepared cells, 1–3 µg of drug resistance cassette DNA, CRISPR mix, and RNAse-free water to a final volume of 45 µL was mixed and transferred to a Bio-Rad Gene Pulser cuvette (0.2-cm gap). Cells were pulsed using an Eppendorf Eporator at 1,500 V and immediately resuspended in 1 mL of ice-cold sorbitol. Cells were collected by centrifugation, resuspended in 1 mL of YPD media, and allowed to recover by incubation at 30°C at 250 rpm for 3–24 hours. Recovered cells were resuspended in 100 µL of YPD and plated onto drug-selective media. Nourseothricin (GoldBio) was used at a final concentration of 300 µg/mL for antibiotic selection of the *NatMX* cassette. Hygromycin B (Cayman) was used at a final concentration of 500 µg/mL. Geneticin (G418, GoldBio) was used at a final concentration of 800 µg/mL. Zeocin (Cayman) was used at a final concentration of 600 µg/mL for *C. glabrata* and 800 µg/mL for *C. auris*. For double selection for *C. albicans* using *SAT1* and *BleMX* or *KanMX*, plates contained 200 µg/mL nourseothricin and 800 µg/mL zeocin or 800 µg/mL G418 sulfate. Colonies were screened via PCR using primers indicated in Table S2. Three independent clones were used for phenotypic characterizations.

### Serial dilution spot assays and liquid growth assays

For serial dilution spot assays, yeast strains were inoculated in SC or YPD media and grown to saturation overnight as previously published (45). Yeast strains were diluted to an OD_600_ of 0.1 and grown in SC or YPD media to log phase with shaking at 30°C. The indicated strains were spotted in fivefold dilutions starting at an OD_600_ of 0.01 on untreated SC or YPD plates or plates containing 8, 16, or 64 µg/mL fluconazole (Cayman). For *C. glabrata*, *C. albicans*, and *C. auris*, plates were grown at 30°C for 48 hours prior to imaging. For liquid growth assays, the indicated yeast strains were inoculated in SC or YPD media and grown to saturation overnight. Yeast strains were diluted to an OD_600_ of 0.1 and grown in SC or YPD media to log phase with shaking at 30°C. The indicated strains were diluted to an OD_600_ of 0.01 in 100 µL SC or YPD media. Cells were left untreated or treated with 20 µg/mL ergosterol (Alfa Aesar) and grown for 50 hours with shaking at 30°C. The OD_600_ was determined every 15 minutes using a Bio-Tek Synergy 4 multimode plate reader.

### Quantitative real-time PCR analysis

RNA was isolated from cells grown in SC media by standard acid phenol purification as previously described (56). ABM All-In-One 5X RT MasterMix (ABM) was used to generate cDNA. Primers for gene expression analysis are indicated in Table S4. A minimum of three biological replicates, as well as three technical replicates, were performed for each biological replicate using the comparative CT method (2^−ΔΔCT^) see Table S5.

### Cell extract and Western blot analysis

Whole cell extraction and Western blot analysis were performed as previously described (57, 58). The anti-HA (Roche 12CA5, 1:10,000) monoclonal antibody was used as previously described (59). Histone H3 rabbit polyclonal antibody (PRF&L) was used at a 1:100,000 dilution as previously described (60).

## SUPPLEMENTAL MATERIAL

Supplemental material msphere.00311-23-s0001.pdf Fig. S1 to S5 and Tables S1 to S5.

ASM does not own the copyrights to Supplemental Material that may be linked to, or accessed through, an article. The authors have granted ASM a non-exclusive, world-wide license to publish the Supplemental Material files. Please contact the corresponding author directly for reuse.

## References

[1] Bongomin F, Gago S, Oladele RO, Denning DW. 2017. Global and multi-national prevalence of fungal diseases—estimate precision. J Fungi (Basel) 3:57. doi:10.3390/jof3040057 29371573 PMC5753159

[2] Rayens E, Norris KA. 2022. Prevalence and healthcare burden of fungal infections in the United States, 2018. Open Forum Infect Dis 9:ofab593. doi:10.1093/ofid/ofab593 35036461 PMC8754384

[3] Borjian Boroujeni Z, Shamsaei S, Yarahmadi M, Getso MI, Salimi Khorashad A, Haghighi L, Raissi V, Zareei M, Saleh Mohammadzade A, Moqarabzadeh V, Soleimani A, Raeisi F, Mohseni M, Mohseni MS, Raiesi O. 2021. Distribution of invasive fungal infections: molecular epidemiology, etiology, clinical conditions, diagnosis and risk factors: a 3-year experience with 490 patients under intensive care. Microb Pathog 152:104616. doi:10.1016/j.micpath.2020.104616 33212195

[4] Pfaller MA, Diekema DJ. 2007. Epidemiology of invasive candidiasis: a persistent public health problem. Clin Microbiol Rev 20:133–163. doi:10.1128/CMR.00029-06 17223626 PMC1797637

[5] Webb BJ, Ferraro JP, Rea S, Kaufusi S, Goodman BE, Spalding J. 2018. Epidemiology and clinical features of invasive fungal infection in a US health care network. Open Forum Infect Dis 5:ofy187. doi:10.1093/ofid/ofy187 30151412 PMC6104777

[6] Rodrigues CF, Silva S, Henriques M. 2014. Candida glabrata: a review of its features and resistance. Eur J Clin Microbiol Infect Dis 33:673–688. doi:10.1007/s10096-013-2009-3 24249283

[7] White TC, Holleman S, Dy F, Mirels LF, Stevens DA. 2002. Resistance mechanisms in clinical isolates of Candida albicans. Antimicrob Agents Chemother 46:1704–1713. doi:10.1128/AAC.46.6.1704-1713.2002 12019079 PMC127245

[8] Rybak JM, Barker KS, Muñoz JF, Parker JE, Ahmad S, Mokaddas E, Abdullah A, Elhagracy RS, Kelly SL, Cuomo CA, Rogers PD. 2022. In vivo emergence of high-level resistance during treatment reveals the first identified mechanism of amphotericin B resistance in Candida auris. Clin Microbiol Infect 28:838–843. doi:10.1016/j.cmi.2021.11.024 34915074 PMC9467277

[9] Satoh K, Makimura K, Hasumi Y, Nishiyama Y, Uchida K, Yamaguchi H. 2009. Candida auris sp. nov., a novel ascomycetous yeast isolated from the external ear canal of an inpatient in a Japanese hospital. Microbiol Immunol 53:41–44. doi:10.1111/j.1348-0421.2008.00083.x 19161556

[10] Johnston EJ, Moses T, Rosser SJ. 2020. The wide-ranging phenotypes of ergosterol biosynthesis mutants, and implications for microbial cell factories. Yeast 37:27–44. doi:10.1002/yea.3452 31800968

[11] Martel CM, Parker JE, Bader O, Weig M, Gross U, Warrilow AGS, Rolley N, Kelly DE, Kelly SL. 2010. Identification and characterization of four azole-resistant erg3 mutants of Candida albicans. Antimicrob Agents Chemother 54:4527–4533. doi:10.1128/AAC.00348-10 20733039 PMC2976150

[12] Alcazar-Fuoli L, Mellado E. 2012. Ergosterol biosynthesis in Aspergillus fumigatus: its relevance as an antifungal target and role in antifungal drug resistance. Front Microbiol 3:439. doi:10.3389/fmicb.2012.00439 23335918 PMC3541703

[13] Jordá T, Puig S. 2020. Regulation of ergosterol biosynthesis in Saccharomyces cerevisiae. Genes (Basel) 11:795. doi:10.3390/genes11070795 32679672 PMC7397035

[14] Watson PF, Rose ME, Ellis SW, England H, Kelly SL. 1989. Defective sterol C5-6 desaturation and azole resistance: a new hypothesis for the mode of action of azole antifungals. Biochem Biophys Res Commun 164:1170–1175. doi:10.1016/0006-291x(89)91792-0 2556119

[15] Sanglard D, Ischer F, Parkinson T, Falconer D, Bille J. 2003. Candida albicans mutations in the ergosterol biosynthetic pathway and resistance to several antifungal agents. Antimicrob Agents Chemother 47:2404–2412. doi:10.1128/AAC.47.8.2404-2412.2003 12878497 PMC166068

[16] Geber A, Hitchcock CA, Swartz JE, Pullen FS, Marsden KE, Kwon-Chung KJ, Bennett JE. 1995. Deletion of the Candida glabrata ERG3 and ERG11 genes: effect on cell viability, cell growth, sterol composition, and antifungal susceptibility. Antimicrob Agents Chemother 39:2708–2717. doi:10.1128/AAC.39.12.2708 8593007 PMC163017

[17] Bhakt P, Raney M, Kaur R. 2022. The SET-domain protein CgSet4 negatively regulates antifungal drug resistance via the ergosterol biosynthesis transcriptional regulator CgUpc2A. J Biol Chem 298:102485. doi:10.1016/j.jbc.2022.102485 36108742 PMC9576903

[18] Akins RA. 2005. An update on antifungal targets and mechanisms of resistance in Candida albicans. Med Mycol 43:285–318. doi:10.1080/13693780500138971 16110776

[19] Ksiezopolska E, Schikora-Tamarit MÀ, Beyer R, Nunez-Rodriguez JC, Schüller C, Gabaldón T. 2021. Narrow mutational signatures drive acquisition of multidrug resistance in the fungal pathogen Candida glabrata. Curr Biol 31:5314–5326. doi:10.1016/j.cub.2021.09.084 34699784 PMC8660101

[20] Pais P, Galocha M, Takahashi-Nakaguchi A, Chibana H, Teixeira MC. 2022. Multiple genome analysis of Candida glabrata clinical isolates renders new insights into genetic diversity and drug resistance determinants. Microb Cell 9:174–189. doi:10.15698/mic2022.11.786 36448018 PMC9662024

[21] Grahl N, Demers EG, Crocker AW, Hogan DA. 2017. Use of RNA-protein complexes for genome editing in non-albicans Candida species. mSphere 2:e00218-17. doi:10.1128/mSphere.00218-17 28657070 PMC5480035

[22] Schwarzmüller T, Ma B, Hiller E, Istel F, Tscherner M, Brunke S, Ames L, Firon A, Green B, Cabral V, Marcet-Houben M, Jacobsen ID, Quintin J, Seider K, Frohner I, Glaser W, Jungwirth H, Bachellier-Bassi S, Chauvel M, Zeidler U, Ferrandon D, Gabaldón T, Hube B, d’Enfert C, Rupp S, Cormack B, Haynes K, Kuchler K. 2014. Systematic phenotyping of a large-scale Candida glabrata deletion collection reveals novel antifungal tolerance genes. PLoS Pathog 10:e1004211. doi:10.1371/journal.ppat.1004211 24945925 PMC4063973

[23] Enkler L, Richer D, Marchand AL, Ferrandon D, Jossinet F. 2016. Genome engineering in the yeast pathogen Candida glabrata using the CRISPR-Cas9 system. Sci Rep 6:35766. doi:10.1038/srep35766 27767081 PMC5073330

[24] Edlind TD, Henry KW, Vermitsky JP, Edlind MP, Raj S, Katiyar SK. 2005. Promoter-dependent disruption of genes: simple, rapid, and specific PCR-based method with application to three different yeast. Curr Genet 48:117–125. doi:10.1007/s00294-005-0008-3 16078083

[25] Vyas VK, Bushkin GG, Bernstein DA, Getz MA, Sewastianik M, Barrasa MI, Bartel DP, Fink GR. 2018. New CRISPR mutagenesis strategies reveal variation in repair mechanisms among fungi. mSphere 3:00154–18. doi:10.1128/mSphere.00154-18 PMC5917429 29695624

[26] Min K, Ichikawa Y, Woolford CA, Mitchell AP, Imperiale MJ. 2016. Candida albicans gene deletion with a transient CRISPR-Cas9 system. mSphere 1:00130–16. doi:10.1128/mSphere.00130-16 PMC4911798 27340698

[27] Goldstein AL, McCusker JH. 1999. Three new dominant drug resistance cassettes for gene disruption in Saccharomyces cerevisiae. Yeast 15:1541–1553. doi:10.1002/(SICI)1097-0061(199910)15:14<1541::AID-YEA476>3.0.CO;2-K 10514571

[28] Lupetti A, Danesi R, Campa M, Del Tacca M, Kelly S. 2002. Molecular basis of resistance to azole antifungals. Trends Mol Med 8:76–81. doi:10.1016/s1471-4914(02)02280-3 11815273

[29] Robbins N, Cowen LE. 2021. Antifungal drug resistance: deciphering the mechanisms governing multidrug resistance in the fungal pathogen Candida glabrata. Curr Biol 31:R1520–R1523. doi:10.1016/j.cub.2021.09.071 34875240

[30] Bhattacharya S, Esquivel BD, White TC, Lorenz M. 2018. Overexpression or deletion of ergosterol biosynthesis genes alters doubling time, response to stress agents, and drug susceptibility in Saccharomyces cerevisiae. mBio 9:e01291-18. doi:10.1128/mBio.01291-18 30042199 PMC6058291

[31] Hitchcock CA, Dickinson K, Brown SB, Evans EG, Adams DJ. 1990. Interaction of azole antifungal antibiotics with cytochrome P-450-dependent 14 alpha-sterol demethylase purified from Candida albicans. Biochem J 266:475–480. doi:10.1042/bj2660475 2180400 PMC1131156

[32] Kelly SL, Lamb DC, Baldwin BC, Corran AJ, Kelly DE. 1997. Characterization of Saccharomyces cerevisiae CYP61, sterol delta22-desaturase, and inhibition by azole antifungal agents. J Biol Chem 272:9986–9988. doi:10.1074/jbc.272.15.9986 9092539

[33] Lamb DC, Maspahy S, Kelly DE, Manning NJ, Geber A, Bennett JE, Kelly SL. 1999. Purification, reconstitution, and inhibition of cytochrome P-450 sterol delta22-desaturase from the pathogenic fungus Candida glabrata. Antimicrob Agents Chemother 43:1725–1728. doi:10.1128/AAC.43.7.1725 10390230 PMC89351

[34] Sun X, Wang W, Wang K, Yu X, Liu J, Zhou F, Xie B, Li S. 2013. Sterol C-22 desaturase ERG5 mediates the sensitivity to antifungal azoles in Neurospora crassa and Fusarium verticillioides. Front Microbiol 4:127. doi:10.3389/fmicb.2013.00127 23755044 PMC3666115

[35] Mount HO, Revie NM, Todd RT, Anstett K, Collins C, Costanzo M, Boone C, Robbins N, Selmecki A, Cowen LE. 2018. Global analysis of genetic circuitry and adaptive mechanisms enabling resistance to the azole antifungal drugs. PLoS Genet 14:e1007319. doi:10.1371/journal.pgen.1007319 29702647 PMC5922528

[36] Liu G, Chen Y, Færgeman NJ, Nielsen J. 2017. Elimination of the last reactions in ergosterol biosynthesis alters the resistance of Saccharomyces cerevisiae to multiple stresses. FEMS Yeast Res 17. doi:10.1093/femsyr/fox063 28910986

[37] Arthington-Skaggs BA, Crowell DN, Yang H, Sturley SL, Bard M. 1996. Positive and negative regulation of a sterol biosynthetic gene (ERG3) in the post-squalene portion of the yeast ergosterol pathway. FEBS Lett 392:161–165. doi:10.1016/0014-5793(96)00807-1 8772195

[38] Skaggs BA, Alexander JF, Pierson CA, Schweitzer KS, Chun KT, Koegel C, Barbuch R, Bard M. 1996. Cloning and characterization of the Saccharomyces cerevisiae C-22 sterol desaturase gene, encoding a second cytochrome P-450 involved in ergosterol biosynthesis. Gene 169:105–109. doi:10.1016/0378-1119(95)00770-9 8635732

[39] Alderton AJ, Burr I, Mühlschlegel FA, Tuite MF. 2006. Zeocin resistance as a dominant selective marker for transformation and targeted gene deletions in Candida glabrata. Mycoses 49:445–451. doi:10.1111/j.1439-0507.2006.01271.x 17022759

[40] Young CL, Raden DL, Caplan JL, Czymmek KJ, Robinson AS. 2012. Cassette series designed for live-cell imaging of proteins and high-resolution techniques in yeast. Yeast 29:119–136. doi:10.1002/yea.2895 22473760 PMC3371369

[41] Cormack BP, Falkow S. 1999. Efficient homologous and illegitimate recombination in the opportunistic yeast pathogen Candida glabrata. Genetics 151:979–987. doi:10.1093/genetics/151.3.979 10049916 PMC1460538

[42] Güldener U, Heck S, Fielder T, Beinhauer J, Hegemann JH. 1996. A new efficient gene disruption cassette for repeated use in budding yeast. Nucleic Acids Res 24:2519–2524. doi:10.1093/nar/24.13.2519 8692690 PMC145975

[43] Bähler J, Wu J-Q, Longtine MS, Shah NG, Mckenzie III A, Steever AB, Wach A, Philippsen P, Pringle JR. 1998. Heterologous modules for efficient and versatile PCR-based gene targeting in Schizosaccharomyces pombe. Yeast 14:943–951. doi:10.1002/(SICI)1097-0061(199807)14:10<943::AID-YEA292>3.0.CO;2-Y 9717240

[44] Chandrasekharan MB, Huang F, Chen YC, Sun ZW. 2010. Histone H2B C-terminal helix mediates trans-histone H3K4 methylation independent of H2B ubiquitination. Mol Cell Biol 30:3216–3232. doi:10.1128/MCB.01008-09 20439497 PMC2897581

[45] Baker KM, Hoda S, Saha D, Gregor JB, Georgescu L, Serratore ND, Zhang Y, Cheng L, Lanman NA, Briggs SD. 2022. The Set1 histone H3K4 methyltransferase contributes to azole susceptibility in a species-specific manner by differentially altering the expression of drug efflux pumps and the ergosterol gene pathway. Antimicrob Agents Chemother 66:e02250–21. doi:10.1128/aac.02250-21 35471041 PMC9112889

[46] Vu BG, Thomas GH, Moye-Rowley WS. 2019. Evidence that ergosterol biosynthesis modulates activity of the Pdr1 transcription factor in Candida glabrata. mBio 10:e00934-19. doi:10.1128/mBio.00934-19 31186322 PMC6561024

[47] Kim SH, Iyer KR, Pardeshi L, Muñoz JF, Robbins N, Cuomo CA, Wong KH, Cowen LE. 2019. Genetic analysis of Candida auris implicates Hsp90 in morphogenesis and azole tolerance and Cdr1 in azole resistance. mBio 10:e00346-19. doi:10.1128/mBio.00346-19 30696744 PMC6355988

[48] Rybak JM, Doorley LA, Nishimoto AT, Barker KS, Palmer GE, Rogers PD. 2019. Abrogation of triazole resistance upon deletion of CDR1 in a clinical isolate of Candida auris. Antimicrob Agents Chemother (Bethesda) 63. doi:10.1128/AAC.00057-19 PMC6437491 30718246

[49] Liu J, Vogel AK, Miao J, Carnahan JA, Lowes DJ, Rybak JM, Peters BM, O’Meara TR. 2022. Rapid hypothesis testing in Candida albicans clinical isolates using a cloning-free, modular, and recyclable system for CRISPR-Cas9 mediated mutant and revertant construction. Microbiol Spectr 10. doi:10.1128/spectrum.02630-21 PMC9241802 35612314

[50] Robbins N, Collins C, Morhayim J, Cowen LE. 2010. Metabolic control of antifungal drug resistance. Fungal Genet Biol 47:81–93. doi:10.1016/j.fgb.2009.07.004 19595784

[51] Park SO, Frazer C, Bennett RJ. 2022. An adjuvant-based approach enables the use of dominant HYG and KAN selectable markers in Candida albicans. mSphere 7:e0034722. doi:10.1128/msphere.00347-22 35968963 PMC9429937

[52] Vyas VK, Barrasa MI, Fink GR. 2015. A Candida albicans CRISPR system permits genetic engineering of essential genes and gene families. Sci Adv 1:e1500248. doi:10.1126/sciadv.1500248 25977940 PMC4428347

[53] Zhang Y, Serratore ND, Briggs SD. 2017. N-ICE plasmids for generating N-terminal 3× FLAG tagged genes that allow inducible, constitutive or endogenous expression in Saccharomyces cerevisiae. Yeast 34:223–235. doi:10.1002/yea.3226 27943405

[54] Fonzi WA, Irwin MY. 1993. Isogenic strain construction and gene mapping in Candida albicans. Genetics 134:717–728. doi:10.1093/genetics/134.3.717 8349105 PMC1205510

[55] Reuss O, Vik A, Kolter R, Morschhäuser J. 2004. The SAT1 flipper, an optimized tool for gene disruption in Candida albicans. Gene 341:119–127. doi:10.1016/j.gene.2004.06.021 15474295

[56] Baker KM, Hoda S, Saha D, Gregor JB, Georgescu L, Serratore ND, Zhang Y, Cheng L, Lanman NA, Briggs SD. 2022. The Set1 histone H3K4 methyltransferase contributes to azole susceptibility in a species-specific manner by differentially altering the expression of drug efflux pumps and the ergosterol gene pathway. Antimicrob Agents Chemother 66:e0225021. doi:10.1128/aac.02250-21 35471041 PMC9112889

[57] Mersman DP, Du HN, Fingerman IM, South PF, Briggs SD. 2012. Charge-based interaction conserved within histone H3 lysine 4 (H3K4) methyltransferase complexes is needed for protein stability, histone methylation, and gene expression. J Biol Chem 287:2652–2665. doi:10.1074/jbc.M111.280867 22147691 PMC3268424

[58] Fingerman IM, Wu CL, Wilson BD, Briggs SD. 2005. Global loss of Set1-mediated H3 Lys4 trimethylation is associated with silencing defects in Saccharomyces cerevisiae. J Biol Chem 280:28761–28765. doi:10.1074/jbc.C500097200 15964832 PMC2745054

[59] South PF, Fingerman IM, Mersman DP, Du HN, Briggs SD. 2010. A conserved interaction between the SDI domain of Bre2 and the Dpy-30 domain of Sdc1 is required for histone methylation and gene expression. J Biol Chem 285:595–607. doi:10.1074/jbc.M109.042697 19897479 PMC2804208

[60] Milholland KL, Gregor JB, Hoda S, Píriz-Antúnez S, Dueñas-Santero E, Vu BG, Patel KP, Moye-Rowley WS, Vázquez de Aldana CR, Correa-Bordes J, Briggs SD, Hall MC. 2023. Rapid, efficient auxin-inducible protein degradation in Candida pathogens. mSphere:e0028323. doi:10.1128/msphere.00283-23 37594261 PMC10597344

