# Supplementary material for: An expanded toolkit of drug resistance cassettes for Candida glabrata, Candida auris, and Candida albicans leads to new insights into the ergosterol pathway
Source: mSphere. 2023 Nov 6;8(6):e00311-23. doi: 10.1128/msphere.00311-23 (PMC10732037; doi:10.1128/msphere.00311-23)
Supplement: Supplemental material — Fig. S1 to S5 and Tables S1 to S5. [file msphere.00311-23-s0001.pdf]

Supplemental Figure 1.

A.

(+) CRISPR-RNP (+) *NatMX* (60 bp HR)	
**WHITE**	**RED**
**108**	**74**
**40% Efficient**	

C.

(+) CRISPR-RNP (+) *NatMX* (60 bp HR)	
**WT**	***erg3Δ***
**28**	**4**
**12% Efficient**	

B.

Organism	Background	Parent Strain	Modified Strain	% Positive Integrants
C. glabrata	*Cg*2001	WT	*erg3Δ NatMX*	56%
C. glabrata	*Cg*2001	WT	*erg3Δ HphMX*	81%
C. glabrata	*Cg*2001	WT	*erg3Δ BleMX*	31%
C. glabrata	*Cg*2001	WT	*erg3Δ KanMX*	75%
C. glabrata	*Cg*2001	WT	*erg5Δ NatMX*	100%
C. glabrata	*Cg*2001	*erg3Δ NatMX*	*erg3Δ NatMX erg5Δ HphMX*	38%
C. glabrata	*Cg*2001	WT	*ERG3*-3xHA *KanMX*	75%
C. glabrata	*Cg*2001	WT	*ERG11*-3xHA *KanMX*	69%
C. auris	*AR0387*	WT	*erg3Δ BleMX*	75%
C. auris	*AR0387*	WT	*erg3Δ KanMX*	75%
C. albicans	SC5314	WT	*erg3Δ SAT1/erg3Δ BleMX*	16%
C. albicans	SC5314	WT	*erg3Δ SAT1/erg3Δ KanMX*	10%

FIG S1 Transformation Efficiencies of *C. glabrata*, *C. auris*, and *C. albicans* with the aid of CRISPR-Cas9 RNP. **(A and C)** Efficiency of *ade2Δ* and *erg3Δ* gene deletions in *C. glabrata* using 60 bp of flanking homologous region (HR) when using CRISPR-Cas9-RNP **(B)** Percentage of positive gene deletions when using 130-150 bp of HR when using CRISPR-Cas9 RNP. Percentages are out of 16 colonies screened for *C. glabrata* and *C. auris* and 32 colonies for *C. albicans*.

Supplemental Figure 2.

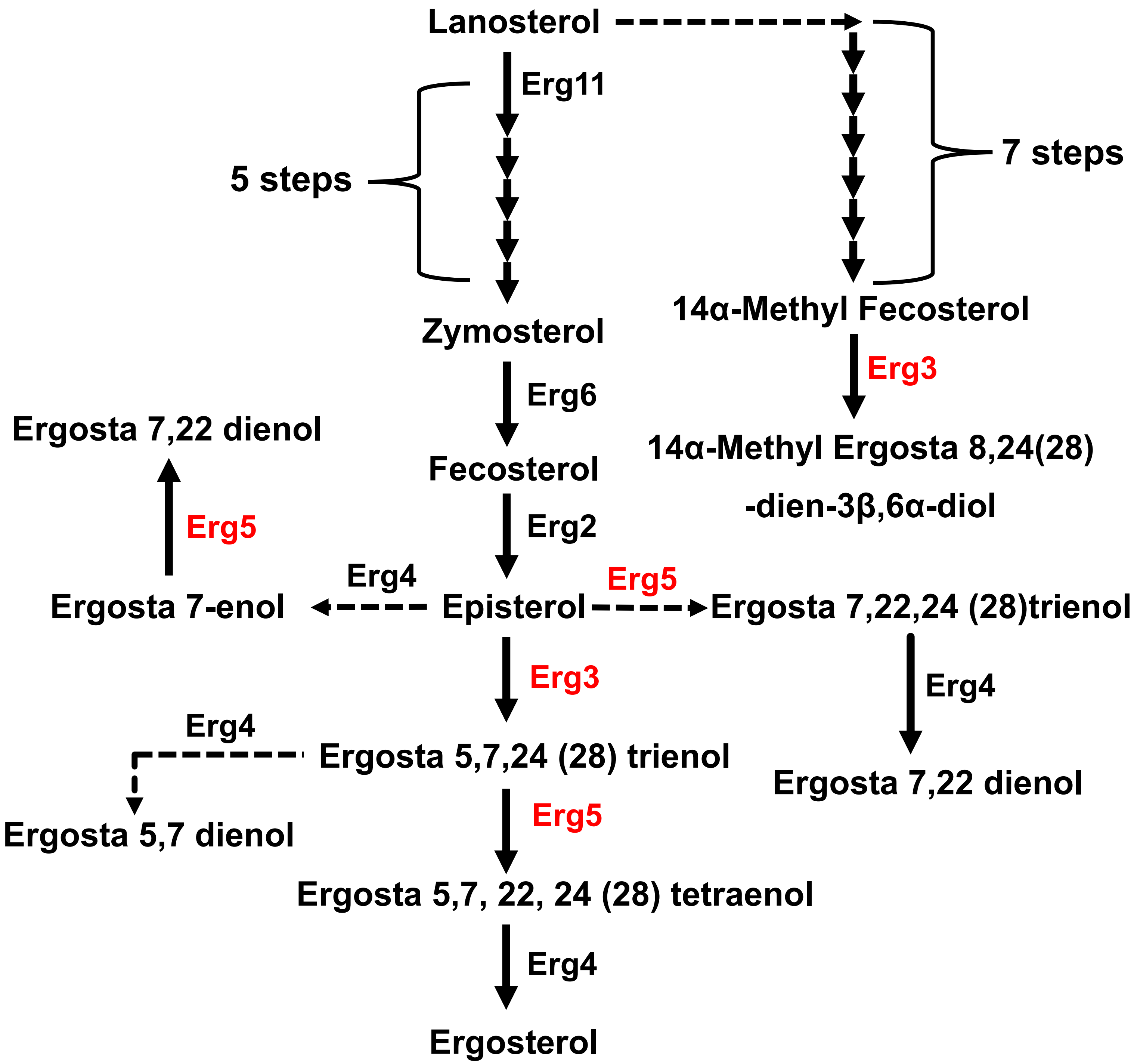

FIG S2 Late Ergosterol Biosynthesis Pathway in *S. cerevisiae* and *C. glabrata*. Azoles inhibit lanosterol 14-α demethylase, leading to the buildup of the Erg3-dependent toxic 14-α methyl ergosta 8,24(28)-dien-3β,6α-diol. Erg3 and Erg5 are shown in red to depict intermediate sterols dependent on either enzyme for synthesis based on known biochemical and genetic data.

Supplemental Figure 3.

A.

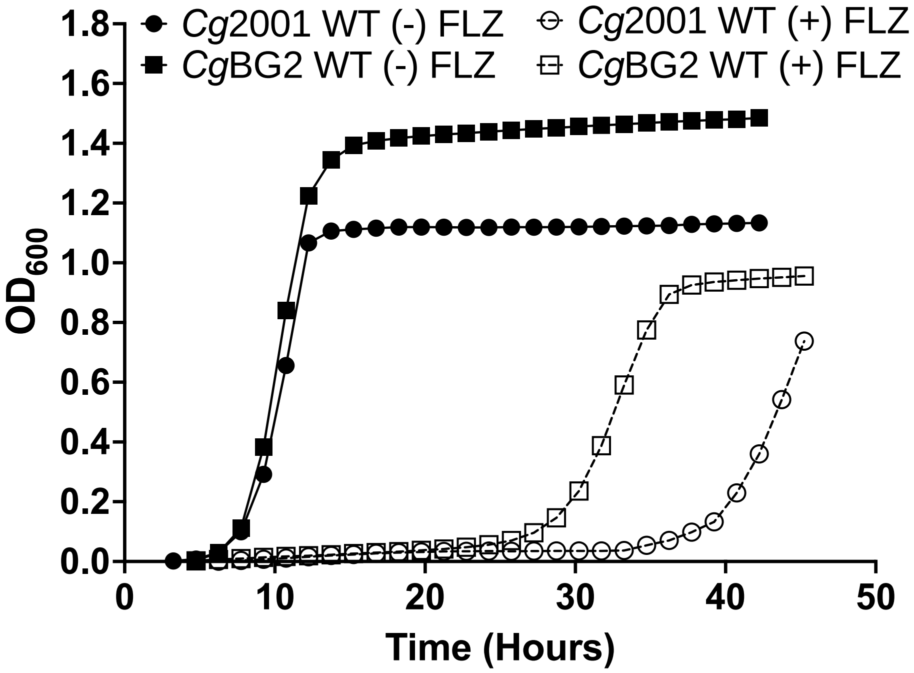

B.

(-) Fluconazole				
**Strain**	**2001 WT**	**2001 *erg3Δ***	**BG2 WT**	**BG2 *erg3Δ***
Doubling Time (mins)	71 ± 4	107 ± 7	62 ± 1	75 ± 7
Time (mins) OD_{600} = 0.5	615 ± 15	735 ± 26	590 ± 31	640 ± 9

C.

(+) Fluconazole				
**Strain**	**2001 WT**	**2001 *erg3Δ***	**BG2 WT**	**BG2 *erg3Δ***
Doubling Time (mins)	150 ± 34	186 ± 12	130 ± 17	223 ± 7
Time (mins) OD_{600} = 0.5	2635 ± 115	1150 ± 38	2010 ± 152	1445 ± 53

FIG S3 *ERG3* deletion results in an azole resistance phenotype in *C. glabrata* in BG2 and *Cg*2001 backgrounds. **(A)** Liquid growth assay of BG2 and *Cg*2001 WT strains with and without 64 µg/mL fluconazole in YPD. Growth curves represent the average of three biological replicates. **(B and C)** Quantified doubling times and time to reach OD_{600} of 0.5 of indicated liquid growth assays with and without 64 µg/mL fluconazole. Numbers represent mean ± SD and are the average of three biological replicates.

Supplemental Figure 4.

(-) Ergosterol				
**Strain**	**WT**	***erg3Δ***	***erg5Δ***	***erg3Δerg5Δ***
Doubling Time (mins)	107 ± 8	204 ± 6	106 ± 7	348 ± 32
Time (mins) OD_{600} = 0.5	435 ± 0	795 ± 26	520 ± 9	1260 ± 212

FIG S4 *ERG* mutants have altered doubling times and lag phases in liquid growth assay in SC media without exogenous ergosterol**.** Quantified doubling times and time to reach OD_{600} of 0.5 of indicated liquid growth assays in SC media in Figure 3D. Numbers represent mean ± SD and are the average of three biological replicates.

Supplemental Figure 5.

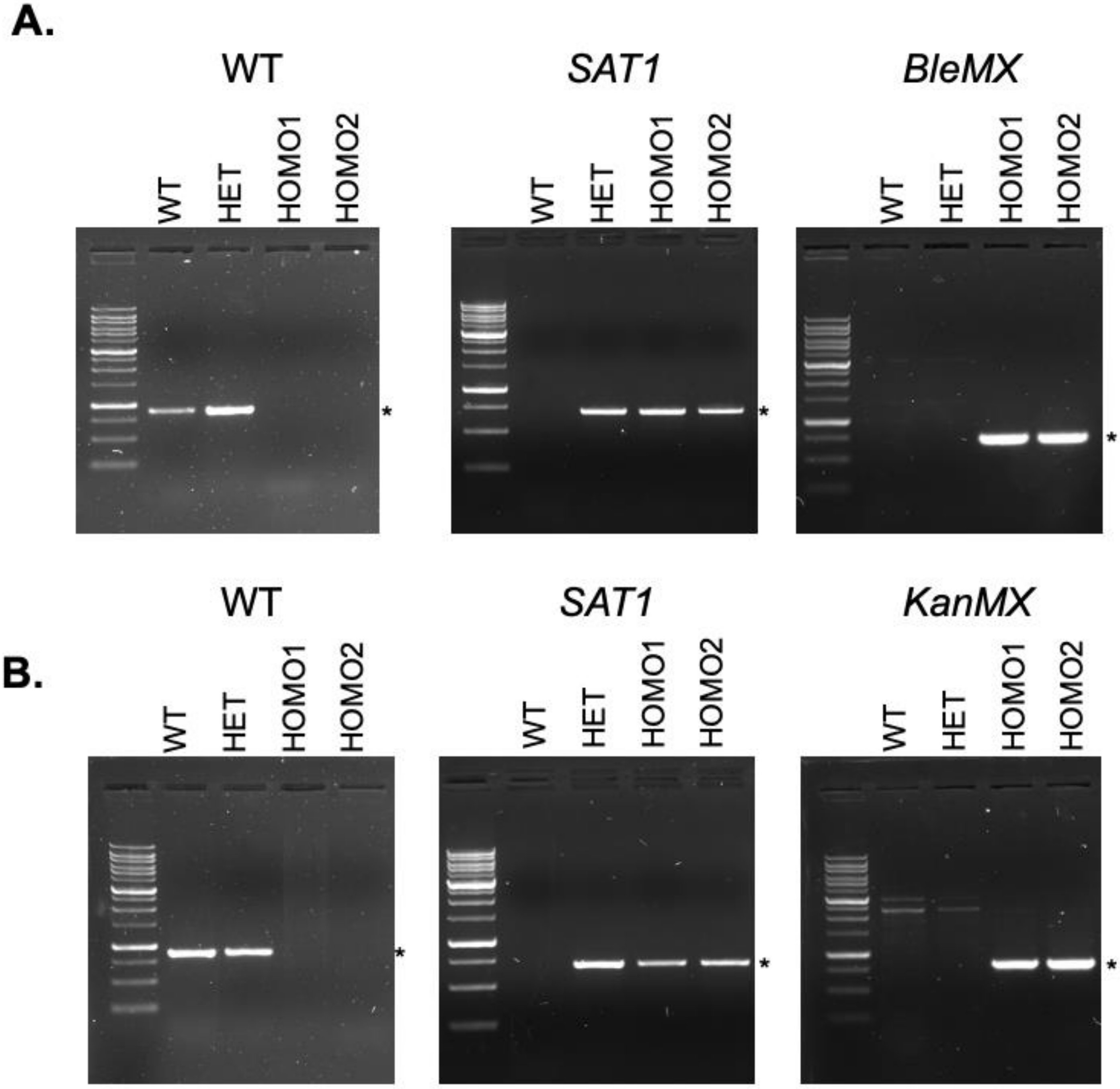

FIG S5 *BleMX* and *KanMX* can be used in conjunction with *SAT1* for homozygous double allele deletions of *ERG3* in the prototrophic *C. albicans* SC5314. **(A)** Agarose gels indicate PCR confirmation of SC5314 WT, *ERG3/erg3Δ* (HET), and two homozygous *erg3Δ/erg3Δ* clones (HOMO1, HOMO2). PCR amplifications of *ERG3* WT specific, *SAT1* insert specific, and *BleMX* insert specific products are shown **(B)** Agarose gels indicate PCR confirmation of SC5314 WT, *ERG3/erg3Δ* (HET), and two homozygous *erg3Δ/erg3Δ* clones (HOMO1, HOMO2). PCR amplifications of *ERG3* WT specific, *SAT1* insert specific, and *KanMX* insert specific products are shown. Asterisks* indicate expected sizes of PCR products.

Supplemental Tables

Table S1: Yeast Strains and Genotype

Yeast Strain	Genotype	Reference	Strain Name
ATCC 2001 *Candida glabrata* WT	*C. glabrata* prototrophic wild type strain	www.atcc.org	*Cg*WT
SDBY1620	*Cg*2001 *erg3Δ::NatMX*	This study	*Cgerg3Δ NatMX*
SDBY1621	*Cg*2001 *erg3Δ::HphMX*	This study	*Cgerg3Δ HphMX*
SDBY1622	*Cg*2001 *erg3Δ::KanMX*	This study	*Cgerg3Δ KanMX*
SDBY1623	*Cg*2001 *erg3Δ::BleMX*	This study	*Cgerg3Δ BleMX*
SDBY1624	*Cg*2001 *erg5Δ::NatMX*	This study	*Cgerg5Δ*
SDBY1625	*Cg*2001 *erg3Δ::NatMX erg5Δ::HphMX*	This study	*Cgerg3Δerg5Δ*
SDBY1626	*Cg*2001 *ERG3-3xHA::KanMX*	This study	*CgERG3*-3xHA
SDBY1627	*Cg*2001 *ERG11-3xHA::KanMX*	This study	*CgERG11*-3xHA
SDBY1632	*Cg*2001 *erg3Δ::ERG3-3xHA::KanMX*	This study	*Cg erg3Δ::ERG3-3xHA*
SDBY1633	*Cg*2001 *erg5Δ::HphMX erg3Δ::ERG3-3xHA::KanMX*	This study	*Cgerg5Δerg3Δ::ERG3-3xHA*
BG2	*C. glabrata* prototrophic wild-type strain	Cormack et al. (1999) Genetics	BG2
SDBY1634	BG2 *erg3Δ::NatMX*	This study	BG2 *erg3Δ*
AR0387	*C. auris* clinical isolate	CDC AR Isolate Bank	*AR0387*
SDBY1630	*AR0387 erg3Δ::BleMX*	This study	*AR0387 erg3Δ BleMX*
SDBY1631	*AR0387 erg3Δ::KanMX*	This study	*AR0387 erg3Δ KanMX*
SC5314	*C. albicans* prototrophic wild type strain	www.attc.org	SC5314
SDBY1635	SC5314 ERG3/*erg3Δ::SAT1*	This study	SC5314 *ERG3/erg3Δ*
SDBY1636	SC5314 *erg3Δ::BleMX/erg3Δ::SAT1*	This study	SC5314 *erg3Δ/erg3Δ SAT1/BleMX*
SDBY1637	SC5314 *erg3Δ::KanMX /erg3Δ::SAT1*	This study	SC5314 *erg3Δ/erg3Δ SAT1/KanMX*

Table S2: Primer Names and Sequences

Name	Sequence
CgERG3-001F KO *NatMX*/*HphMX*	ctgggcccatacgaccgttacataattgcccagtgcagccatcggtttttacctatacgtgggaactacgagaacaagagctaagagtataaatattgggtacatttgtcttgcatttcagataacctacagccagtagaag**CGCCAGATCTGTTTAGCTTGCCTTGTCC**
CgERG3-002R KO *NatMX*/*HphMX*	gggtcatgaaagagttatgatgtaggaaaagtaatgtgtgcgcgagacaccggtgtttcctgtctagttggtcttcttcttgtcggtgttgtagactctgtctgtgtcgtcaccttccacctcttggatgtaagcggcggtttctctagcttgc**GTGGATCTGATATCATCGATGAATTCG**
CgERG3-003F KO *KanMX*	ctgggcccatacgaccgttacataattgcccagtgcagccatcggtttttacctatacgtgggaactacgagaacaagagctaagagtataaatattgggtacatttgtcttgcatttcagataacctacagccagtagaag**CAGCTGAAGCTTCGTACG**
CgERG3-004R KO *KanMX*	gggtcatgaaagagttatgatgtaggaaaagtaatgtgtgcgcgagacaccggtgtttcctgtctagttggtcttcttcttgtcggtgttgtagactctgtctgtgtcgtcaccttccacctcttggatgtaagcggcggtttctctagcttgc**CATAGGCCACTAGTGGATCTG**
CgERG3-005F 3xHA Tag *KanMX*	gaggactccctgttcgaccctaagctaaagatggacaagaaggtcctagaaaagcaagctagagaaaccgccgcttacatccaagaggtggaaggtgacgacacagacagagtctacaacaccgacaagaagaagaccaac**ATCTTTTACCCATACGATGTTCT**
CgERG3-006R 3xHA Tag *KanMX*	gagaagtaatgcgcatcgcagatcaaaggttggtaagccatagctgcatacgatgattggtgtaggagggtcagtacatgataaagaggaccgttatttgagtaaatagttcttgagtgaacttctatttcatattggtatat**ATCGATGAATTCGACGTCG**
CgERG3-007F Verification Primer	agaagagctgatctctctagaagtg
CgERG3-008F Verification Primer	acacatgtccaacaacccag
CgERG3-009R Verification Primer	tgtggaggcgaggagtagaaag
CgERG3-010F KO *BleMX*	ctgggcccatacgaccgttacataattgcccagtgcagccatcggtttttacctatacgtgggaactacgagaacaagagctaagagtataaatattgggtacatttgtcttgcatttcagataacctacagccagtagaag**CCAGATCTGTTTAGCTTGCCTC**
CgERG3-011R KO *BleMX*	gggtcatgaaagagttatgatgtaggaaaagtaatgtgtgcgcgagacaccggtgtttcctgtctagttggtcttcttcttgtcggtgttgtagactctgtctgtgtcgtcaccttccacctcttggatgtaagcggcggtttctctagcttgc**GCCACTAGTGGATCTGATATCATC**
CgERG5-001F KO *NatMX*/*HphMX*	actggcttctgctaaacgagcgattgttcgtcctcgtaatctcgtaatctcgtaatgcggttcttccggtgaacccggtcgctaacgcaaattttctgaataatataaatacggctagtgcaattgggatacccgattcccttctcttacaggaaaagcaaacttcaccc**CGCCAGATCTGTTTAGCTTGCCTTGTCC**
CgERG5-002R KO *NatMX*/*HphMX*	caatggagtgaccttgtgcttgaagtcagtgaatagagcgaacttaccgatcaaagcagtcatggtaatcatgacataggtttgacctagacaaacgtgtggaccacagccgaagaccaac

	cagttcttcttagcttcgttagctggagaaccttcgac**GTGGATCTGATATCATCGATGAATTCG**
CgERG5-003F Verification Primer	cgcggatttctgagagagagg
CgERG11-001F 3xHA Tag *KanMX*	cgcttactgtcaattgggtgtgttgatgtccattttcatcagaaccatgaaatggcgttacccaactgaaggtgaaactgtcccaccatctgacttcacctccatggtcaccctaccaactgcccctgctaagatctactgggaaaagagacatccagaacaaaagtac**ATCTTTTACCCATACGATGTTCT**
CgERG11-002R 3xHA Tag *KanMX*	ctgcatacattgctagttatacggatgaagacatcgtatagttcgtttagcagcaaagccctctaaacgaaacaaccagcttaagtcatgccgaaatatccatgttgatattcacgatgacttactattaggctaatgaatcagcgtatatcccgtatacgagccagacagc**ATCGATGAATTCGACGTCG**
CgERG11-003F Verification Primer	gtggtggtagacacagatgtatcg
CgERG11-004R Verification Primer	ggaaatataaacaacatgggcgc
AgTEF1-001R Verification Primer	ggaagtatatgaaagaagaacctcagtgg
CgADE2-001F KO *NatMX*/*HphMX*	gtgtcaagttccaattgagagatcagaccctttcaaaaaccactaaaacggtttgataatattacttgtgtgaagctactgtcatcagtttctatcagattcatttgttaccaacgatacaggtttattttgcttacgaataata**CGCCAGATCTGTTTAGCTTGCCTTGTCC**
CgADE2-002R KO *NatMX*/*HphMX*	catatcacgaagaaattttgcattaaatatattatagcatattatgtaaacagcaatagacatttaaaaacatatgaagttaagatacataaatcttctgaatttcaagcaaagactaactggttttatagatggtgct**GTGGATCTGATATCATCGATGAATTCG**
CgADE2-003F KO *BleMX*	gtgtcaagttccaattgagagatcagaccctttcaaaaaccactaaaacggtttgataatattacttgtgtgaagctactgtcatcagtttctatcagattcatttgttaccaacgatacaggtttattttgcttacgaataatac**CAGATCTGTTTAGCTTGCCTC**
CgADE2-004R KO *BleMX*	catatcacgaagaaattttgcattaaatatattatagcatattatgtaaacagcaatagacatttaaaaacatatgaagttaagatacataaatcttctgaatttcaagcaaagactaactggttttatagatggtgct**GCCACTAGTGGATCTGATATCATC**
CgADE2-005F KO *KanMX*	gtgtcaagttccaattgagagatcagaccctttcaaaaaccactaaaacggtttgataatattacttgtgtgaagctactgtcatcagtttctatcagattcatttgttaccaacgatacaggtttattttgcttacgaataata**CAGCTGAAGCTTCGTACG**
CgADE2-006R KO *KanMX*	catatcacgaagaaattttgcattaaatatattatagcatattatgtaaacagcaatagacatttaaaaacatatgaagttaagatacataaatcttctgaatttcaagcaaagactaactggttttatagatggtgct**CATAGGCCACTAGTGGATCTG**

CaurERG3-001F KO *BleMX*	ctattcatagttccagtattgaccccacattggctgtgtattcccccgaaacaatgttccccttaccggcatacaaacaaagaaaccgagtcggacgatgctcgtttagagctcgttttcagtatacgaggc**CAGATCTGTTTAGCTTGCCTC**
CaurERG3-002R KO *BleMX*	cgggccttctgtacgagcggcccaatctgtcccacaacgtggtgaattggccatagttgtagttgaagtacaagtggtgcacagtgtggcacgctgtgccattgaccacggggtcattcgacatgtactggccg**GGCGGCGTTAGTATCGAATC**
CaurERG3-003F Verification Primer	cacggattagacagtttaccgg
CaurERG3-004R Verification Primer	gccacggaaaagtaaagaatccac
CaurERG3-005F KO KanMX	ctattcatagttccagtattgaccccacattggctgtgtattcccccgaaacaatgttccccttaccggcatacaaacaaagaaaccgagtcggacgatgctcgtttagagctcgttttcagtatacgagg**GATGATTCATCCCATTCATTCCATC**
CaurERG3-006R KO KanMX	cgggccttctgtacgagcggcccaatctgtcccacaacgtggtgaattggccatagttgtagttgaagtacaagtggtgcacagtgtggcacgctgtgccattgaccacggggtcattcgacatgtactggccg**GCCGCTCTAGAACTAGTGGATCT**
CaurADE2-001F KO BleMX	gaattcttggaggcggccagttggggcggatgattgttgaggctgcacacagactcaacattaaaactgtggtgcttgacgctgctcattctccagccaagcagatcaacgctttggacgaccatgtggacggctcattc**CCAGATCTGTTTAGCTTGCCTC**
CaurADE2-002R KO BleMX	tcagtatttttcgtagcccacgctttctagtctctcagctttttgcaagacttcctcttccatgttgttcatatattgggacatttcatctaaccacttgtagtcataggctcccaaaatacgaatggccaacaagg**GGCGGCGTTAGTATCGAATC**
CaurADE2-003F KO KanMX	gaattcttggaggcggccagttggggcggatgattgttgaggctgcacacagactcaacattaaaactgtggtgcttgacgctgctcattctccagccaagcagatcaacgctttggacgaccatgtggacggctcattc**GATGATTCATCCCATTCATTCCATC**
CaurADE2-004R KO KanMX	tcagtatttttcgtagcccacgctttctagtctctcagctttttgcaagacttcctcttccatgttgttcatatattgggacatttcatctaaccacttgtagtcataggctcccaaaatacgaatggccaacaagg**GCCGCTCTAGAACTAGTGGATCT**
CaERG3-001F KO *SAT1*/Ca*KanMx*	gttaatccttgctcattatcatatcttgacctaagattcctacaatctagatatctttggacattctattcccttcccatttctttccctattgtgcatataagttcaatctttttttctttctttcggattcggtttagctaattttactacc**GGGTACCGGGCCC**
CaERG3-002R KO *SAT1*/Ca*KanMx*	catcaaatctcaaaattttaaactcaaactcaaacttaaacttaaaaaaaaataaaacaaaaacaaaaacgaaaaataaaaataaaataaaataaaatatctatatcatcaaaattggaaaaatagtcaatggtccaaaacaaagatgtaccaa**GCCGCTCTAGAACTAGTGG**
CaERG3-003F KO *BleMX*	gttaatccttgctcattatcatatcttgacctaagattcctacaatctagatatctttggacattctattcccttcccatttctttccctattgtgcatataagttcaatctttttttctttctttcggattcggtttagctaattttactacc**CCAGATCTGTTTAGCTTGCCTC**

CaERG3-004R KO *BleMX*	catcaaatctcaaaattttaaactcaaactcaaacttaaacttaaaaaaaaataaaacaaaaacaaaaacgaaaaataaaaataaaataaaataaaatatctatatcatcaaaattggaaaaatagtcaatggtccaaaacaaagatgtaccaa**GGCGGCGTTAGTATCGAATC**
CaERG3-005F Verification Primer	ggagtggtgttactttcgtttaag
CaERG3-006R Verification Primer	ggtccaaaacaaagatgtacc
SAT1-001R Verification Primer	cccactccccaatctttgaat
CaKAN-001F Verification Primer	gatggtcagacttaactggc

*Bold and underlined sequences indicate amplification sequences; KO = Knock Out primers; Verification = primers used to confirm deletions and epitope tag strains

Table S3: CRISPR gRNA sequences

**Name**	**Sequence**
CgERG3 gRNA-001 KO	ataccacgttgcaactcaac**AGG**
CgERG3 gRNA-002 KO	acacatgtccaacaacccag**TGG**
CgERG3 gRNA-003 C-Terminal Tag	cctttttgatagttttgcaa**AGG**
CgERG5 gRNA-001 KO	ttcaaagtctaccctatcat**CGG**
CgERG5 gRNA-002 KO	accagactaccaagctccaa**AGG**
CgERG11 gRNA-001 C-Terminal Tag	aggttttgaccattgattat**TGG**
CgADE2 gRNA-001 KO	cgacaatcatacggcccaac**TGG**
CgADE2 gRNA-002 KO	agactatagtgacctcaaat**GGG**
pAG25 *NatMX* gRNA	ctcttgacgacacggcttac**CGG**
CaERG3 gRNA-001 KO	gaatccaaatttgggagact**TGG**
CaurERG3 gRNA-001 KO	gtactaactgcaattcacca**TGG**
CaurERG3 gRNA-002 KO	tgtgcaacgggaacaccatg**GGG**

CaurADE2 gRNA-001 KO	aggatgtcgatgctatcacc**AGG**
CaurADE2 gRNA-002 KO	ggaacaccaatgacaggcaa**AGG**

*Bold and underlined sequences indicate PAM sequence; KO = Knock Out primers

Table S4: qRT-PCR Primers used for *ERG3*, *ERG5* and *RDN18* expression analysis

Name	Sequence
CgERG3 F GE	tgggagcaccacggtctaag
CgERG3 R GE	cagtcggtgaagaagatgaaagtg
CgERG5 F GE	gtcaccgccgctttgg
CgERG5 R GE	ccgtaccaggtcttggtgaaa
CgRDN18-001F	acggagccagcgagtctaac
CgRDN18-002R	cgacggagtttcacaagattacc

*GE = Gene expression primers

Table S5: qRT-PCR analysis of *ERG3* and *ERG5* gene expression

Gene	Strain	Mean RQ	St. Dev	N	p-value
CgERG3	*Cg*WT	1.00	0.42	3	N/A
CgERG3	*Cgerg3Δ*	0.00 (n.e)	0.00	3	0.01
CgERG3	*Cgerg3Δerg5Δ*	0.00 (n.e)	0.00	3	0.01
CgERG3	*Cgerg5Δ*	0.38	0.22	3	0.08
CgERG5	*Cg*WT	1.00	0.61	3	N/A
CgERG5	*Cgerg3Δ*	2.17	0.79	3	0.10
CgERG5	*Cgerg3Δerg5Δ*	0.00 (n.e)	0.00	3	0.04
CgERG5	*Cgerg5Δ*	0.00 (n.e)	0.00	3	0.04

n.e. = not expressed or no detectable expression in deletion strains
